# Enhanced *Synechococcus* Growth Under Extended High-Light and High-Temperature Stress by the F_1_-α-C252Y Mutation in ATP Synthase: ATP Generation and Metabolic Network Remodeling

**DOI:** 10.3390/md24050152

**Published:** 2026-04-25

**Authors:** Linan Zhou, Wenjing Lou, Xin Guo, Siyan Yi, Wenhui Lou, Guodong Luan, Xuefeng Lu

**Affiliations:** 1 College of Life Sciences, Qingdao Agricultural University, Qingdao 266109, China; zhouln@qibebt.ac.cn; 2 Key Laboratory of Photoelectric Conversion and Utilization of Solar Energy, Qingdao Institute of Bioenergy and Bioprocess Technology, Chinese Academy of Sciences, Qingdao 266101, China; guoxin@qibebt.ac.cn (X.G.); cynthiayisy@outlook.com (S.Y.); luangd@qibebt.ac.cn (G.L.); 3 Shandong Energy Institute, Qingdao 266101, China; 4 Qingdao New Energy Shandong Laboratory, Qingdao 266101, China; 5 School of Life Sciences, Henan University, Kaifeng 475004, China; 6 Hunan Provincial Key Laboratory of Forestry Biotechnology, Central South University of Forestry and Technology, Changsha 410004, China; 7 School of Life Sciences, Xinyang Normal University, Xinyang 464000, China; l473973649@foxmail.com

**Keywords:** cyanobacteria, ATP synthase, high-light–high-temperature stress, mutation

## Abstract

Photosynthesis, the main energy source for life on Earth, confronts escalating challenges of high-light–high-temperature stress (HLHT). Our previous study identified a mutation in ATP synthase, F_1_-α-C252Y, that significantly enhances the HLHT tolerance of *Synechococcus elongatus* PCC 7942 (*Sye7942*), although the underlying mechanism remains obscure. In this study, we found that this mutation led to elevated levels of the b subunit of F_o_, F_1_ subunits, and the ATP synthase within cells, without affecting ATP synthetic activity, indicating improved intracellular ATP synthesis activity. Additionally, the mutation altered the transcriptome of *Sye7942*, impacting the expression of genes involved in crucial processes, such as the electron transport chain, carbon fixation, and regulatory factors, which are crucial for cyanobacteria’s adaptation to stresses. Correspondingly, the mutant exhibited enhanced photosynthesis, accelerated growth, and increased glycogen under HLHT conditions, showing improved adaptation. The higher intracellular ATP synthesis activity, along with enhanced photosynthetic activity, suggests increased ATP production in the mutant under HLHT. Enhancing ATP production and remodeling the cellular transcriptome appear to be key strategies employed by the C252Y mutation for *Sye7942* acclimating to HLHT. These findings provide valuable insights for enhancing photosynthetic efficiency and stress resilience in cyanobacteria and other photosynthetic organisms facing HLHT challenges.

## 1. Introduction

Photosynthesis is a critical process on Earth that harnesses solar energy and carbon dioxide to generate chemical energy and carbohydrates [1]. It serves as the main source of food and energy for all life forms. Light, as the primary energy source for photosynthesis, is absorbed by the photosystems, leading to charge separation in the photoreaction centers [2]. The electron transport chain converts light energy into transmembrane proton motive force (PMF), which drives ATP synthase to produce ATP and NADPH, supporting carbon fixation and other cellular metabolic processes. However, excessive light intensity can lead to photodamage when it surpasses the photosynthetic capacity [3]. High light levels in conjunction with elevated temperatures, commonly seen in nature, exacerbate the negative impacts on photosynthetic organisms. This leads to reduced photosynthetic activity, triggering photoinhibition and photobleaching, ultimately limiting their efficiency [4,5,6]. The escalating global temperatures caused by greenhouse gas emissions have worsened these challenges, highlighting the need to boost the resilience of photosynthetic organisms to withstand the intensified high-light–high-temperature (HLHT) stress. This issue has received significant attention. It is critical to enhance the resilience of photosynthetic organisms against HLHT to ensure the continued supply of clean energy and healthy food, promoting societal advancement in the modern era [7,8].

Cyanobacteria, the earliest oxygenic photosynthetic organisms and ancestral to chloroplasts in plants and eukaryotic algae, play a pivotal role in the investigation and understanding of photosynthetic mechanisms. Our group previously reported that a single amino acid substitution at F_o_F_1_ ATP synthase subunit α (AtpA), C252Y, significantly enhances the resistance of *Synechococcus elongatus* PCC 7942 (*Sye*7942) to HLHT [5,9]. The mutant exhibits an accelerated growth rate and increased accumulation of organics under HLHT conditions in comparison to the wild-type. Conversely, the wild-type is unable to survive under the same HLHT conditions. The C252Y mutation results in elevated levels of AtpA protein, intracellular ATP synthetic activity, and ATP abundance, providing the energy needed for repairing photodamage, protecting cells from stress during short periods (up to 4 h) of HLHT [9]. However, the precise effects of the C252Y mutation on ATP synthase and the mechanisms underlying its significant enhancement of photosynthetic efficiency during constant HLHT conditions are still unknown.

ATP synthase consists of two subcomplexes, F_1_ (α_3_β_3_γεδ) and F_o_ (ac_n_bb’). The AtpA and β (AtpB) subunits are arranged in an alternating pattern, forming a hexameric structure with the catalytic site situated at their interface [10]. This enzyme uses a transmembrane PMF to convert ADP and Pi into ATP, the main energy source for cellular processes. As a pivotal photosynthetic membrane complex and energy producer, ATP synthase significantly influences photosynthetic efficiency and stress adaptation in photosynthetic organisms [10]. Many studies have indicated that elevating cellular ATP synthase activity can enhance photosynthetic activity, cellular ATP levels, and carbon fixation [11,12,13,14]. Overexpressing the AtpA or AtpB subunits of ATP synthase has been shown to enhance the resilience of photosynthetic organisms to various stressors [11,12]. Moreover, some subunits of ATP synthase are involved in gene regulation at both the transcription and translation levels, thereby modulating the metabolic network and contributing to stress resistance [15,16,17]. Overall, there are several potential mechanisms through which ATP synthase may contribute to the stress resistance of photosynthetic organisms.

To explore how the C252Y mutation enhances *Sye*7942 adaptability to exposure to constant HLHT, this study investigated changes in ATP synthetic activity, ATP synthase complex and its individual subunit levels, ATP content, photosynthetic activity, reactive oxygen species (ROS), carbohydrate content, and gene expression in both the wild-type and mutant strains under HLHT and normal temperature normal light (NTNL) conditions. Results showed that the C252Y mutation heightened intracellular ATP synthesis activity by increasing ATP synthase content and altering the overall transcriptomes, restructuring the metabolic network under HLHT. These alterations improved cyanobacterial solar energy conversion efficiency and supported their adaptation to challenging environments. This underscores the essential role of the C252 site and AtpA subunit in regulating the intracellular ATP synthase levels and gene expression for cyanobacterial survival in challenging HLHT conditions.

## 2. Results

### 2.1. Determining the Optimal High-Light–High-Temperature Conditions for Growth of the Wild-Type and C252Y Mutant Strains

**Figure 1:**
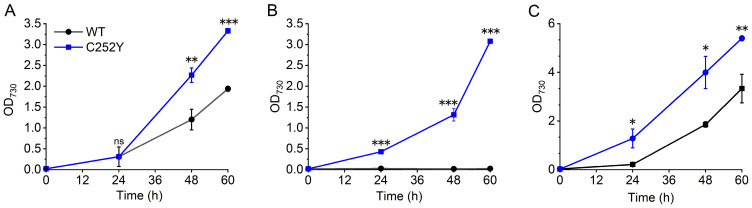
Growth of *Sye*7942 strains under various light and temperature conditions. The wild-type and C252Y mutant strains were cultured in liquid BG11 medium at (**A**) 30 °C with 280 μmol photons m^−2^ s^−1^ illumination (NTNL); (**B**) 42 °C with 2000 μmol photons m^−2^ s^−1^ illumination; and (**C**) 40 °C with 2000 μmol photons m^−2^ s^−1^ illumination (HLHT). WT, wild-type *Sye*7942; C252Y, the C252Y mutant. Star, significant differences (*p* < 0.05); two stars, highly significant (*p* < 0.01); three stars, extremely significant (*p* < 0.001); and ns, no significant difference. Error bars indicate standard deviations (n = 3).

The wild-type and mutant strains were cultivated in the MC-1000 cultivator, maintaining precise light intensity and temperature control for the cultures. Under NTNL conditions (30 °C, 280 μmol photons m^−2^ s^−1^), the mutant displayed superior growth compared to the wild-type (Figure 1A). Conversely, the wild-type strain struggled to survive in 42 °C, 2000 μmol photons m^−2^ s^−1^ conditions, whereas the mutant exhibited accelerated growth rates under the same conditions (Figure 1B). To investigate the effects of the C252Y mutation during constant HLHT, we further evaluated the characteristics of these two strains under more moderate HLHT settings (41 °C, 2000 μmol photons m^−2^ s^−1^; 41 °C, 1500 μmol photons m^−2^ s^−1^; and 40 °C, 2000 μmol photons m^−2^ s^−1^) to obtain ample biomass for further analysis. Under all these HLHT conditions, the mutant demonstrated rapid growth rates (Figure 1, Supplemental Figure S1). While the wild-type strain struggled at 41 °C, 2000 μmol photons m^−2^ s^−1^ conditions (Figure S1A), it exhibited robust growth under 41 °C, 1500 μmol photons m^−2^ s^−1^ conditions without significant differences compared to the mutant (Supplemental Figure S1B). At 40 °C, 2000 μmol photons m^−2^ s^−1^ conditions, the wild-type displayed rapid growth, slower than the mutant (Figure 1C). Therefore, we selected 40 °C, 2000 μmol photons m^−2^ s^−1^ as the HLHT conditions for subsequent analysis.

### 2.2. The Effects of the C252Y Mutation on the Intracellular Levels of ATP Synthase Subunits

**Figure 2:**
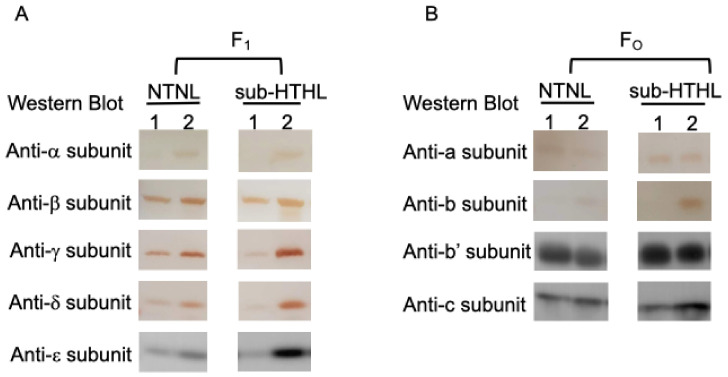
The cellular protein levels of the F_1_ (**A**) and F_o_ (**B**) complex subunits of ATP synthase in the wild-type and C252Y mutant strains under NTNL (30 °C, 280 μmol photons m^−2^ s^−1^) and HLHT (40 °C, 2000 μmol photons m^−2^ s^−1^) conditions. Western blot analysis was used to determine protein levels, with samples containing 1.6 μg chlorophyll a resolved by gradient SDS-PAGE (4–20%), transferred to nitrocellulose membranes, and incubated with specific antibodies. Protein bands were visualized using the BCIP/NBT color development kit (SW1020, Solarbio) for the α, β, γ, δ, a, and b subunits and the Chemiluminescent EMSA Kit (GS009, Beyotime) for the ε, b’, and c subunits to prevent interference from photosynthetic pigments. Coomassie Brilliant Blue-stained SDS-PAGE was used to visualize the normalized protein samples (Supplemental Figure S2). Line 1, wild-type *Sye*7942; line 2, the C252Y mutant. The original figures were presented in Supplemental Figures S3–S10.

Differences in the levels of ATP synthase subunits were analyzed in the wild-type and C252Y mutant under both NTNL and HLHT conditions. In Figure 2A, the AtpA protein content was notably higher in the mutant compared to the wild-type under both NTNL and HLHT conditions, consistent with our prior research [9]. Furthermore, the other subunits of the F_1_-subcomplex—including β, γ, ε, and δ—showed similar increased protein levels in the mutant compared to the wild-type under both NTNL and HLHT conditions. In the mutant strain, all F_1_-subcomplex subunits, except for the AtpB subunit, displayed higher protein levels under HLHT conditions, while in the wild-type strain, these levels were notably lower compared to NTNL conditions (Figure 2A and Supplemental Table S1). Interestingly, the AtpB subunit showed similar protein levels under both NTNL and HLHT conditions in both the wild-type and mutant strains. The protein content of all F_o_-subcomplex subunits, except for the subunit b, remained relatively consistent in the two strains under both NTNL and HLHT conditions. However, under HLHT conditions, there was an increase in protein content for both strains (Figure 2B and Supplemental Table S1). Notably, subunit b’s pattern of changes aligned with those observed in the F_1_-subcomplex subunits, showing a significant increase in the mutant strain under both NTNL and HLHT conditions.

### 2.3. The Influence of the C252Y Mutation on the Intracellular Abundance of ATP Synthase and Its Impact on ATP Synthesis Activity

**Figure 3:**
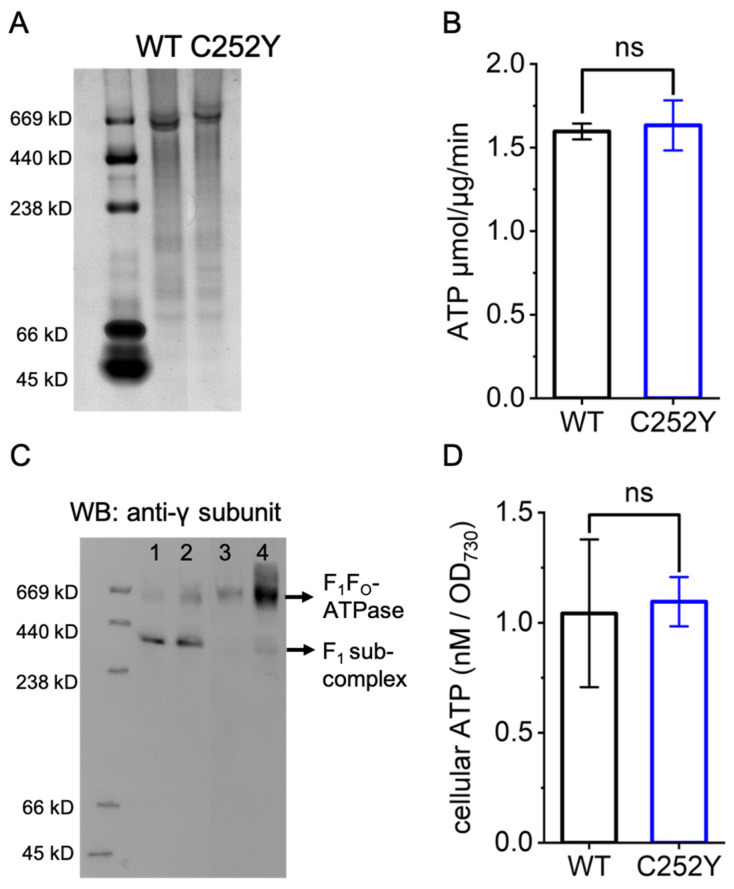
ATP synthetic activity and intracellular levels of ATP synthase. (**A**) The quality of cyanobacterial wild-type and C252Y mutant ATP synthase purified from *E. coli* was assessed using stained Native-PAGE (4–20%). (**B**) ATP synthetic activity analysis of the wild-type and C252Y mutant ATP synthase. (**C**) Protein levels of the F_1_ and ATP synthase complex in the wild-type and C252Y mutant strains were assessed under NTNL (30 °C, 280 μmol photons m^−2^ s^−1^) and HLHT (40 °C, 2000 μmol photons m^−2^ s^−1^) conditions using immunoblot analysis with an antibody targeting the γ subunit. The complete cellular protein samples were separated by gradient Native-PAGE (4–20%). (**D**) Intracellular ATP levels in the wild-type and C252Y mutant under HLHT conditions. WB, Western blot; WT, wild-type; C252Y, the C252Y mutant. Lines 1 and 3, wild-type Sye7942; lines 2 and 4, the C252Y mutant; lines 1 and 2, grown under NTNL conditions; lines 3 and 4, grown under HLHT conditions. Ns, no significant difference (n > 3). Coomassie Brilliant Blue-stained SDS-PAGE was used to visualize the normalized protein samples (Supplemental Figure S2). The original Figure 3C refers to Supplemental Figure S11.

In ATP synthase, the catalytic site is found at the junction of the AtpA and AtpB subunits, with the AtpB subunit making a significant contribution [18]. It has been reported that the C252Y mutation is not located at the interface of the AtpA and AtpB subunits, nor in the recognized functional sites of the AtpA subunit [9]. The impact of the mutation on the activity of ATP synthase is currently still under discussion [19]. To investigate the functional significance of the C252Y mutation in ATP synthase, the wild-type and C252Y mutation ATP synthase from *Sye*7942 were expressed in *Escherichia coli* (*E. coli*) DK8 strain (*Δ*ATPase), purified using affinity chromatography, and their activities were assessed using the acid–base transition method as described previously [9]. The purified ATP synthase complex remained intact (Figure 3A), and there was no significant difference in ATP synthesis activity between the wild-type and C252Y mutant ATP synthase (Figure 3B). This aligns with the prior findings that introducing the mutation F_1_-α-Y252C into ATP synthase of *Synechocystis* PCC 6803 did not alter the ATP synthesis activity of the enzyme [20].

To further explore the impact of the C252Y mutation, the cellular levels of ATP synthase complex were evaluated in the wild-type and C252Y mutant strains under both NTNL and HLHT conditions. The results suggest a noticeable increase in ATP synthase levels in the mutant strain compared to the wild-type, with a more pronounced enhancement observed under HLHT conditions (Figure 3C and Supplemental Table S1). Additionally, the cellular F_1_-subcomplex demonstrated a significant decrease in the two strains under HLHT conditions compared to NTNL conditions (Figure 3C and Supplemental Table S1). The increased intracellular ATP synthase complex levels with unchanged ATP synthesis activity indicate a higher intracellular ATP synthesis activity in the mutant. Interestingly, there was no difference in intracellular ATP levels between the wild-type and C252Y mutant under constant HLHT conditions (Figure 3D).

### 2.4. The Effect of the C252Y Mutation on Intracellular Gene Transcription and Photosynthetic Physiology

In addition to its importance in energy production in photosynthetic organisms, ATP synthase likely directly influences cellular gene transcription and translation in these organisms [15,16,17]. Moreover, introducing the C252Y mutation into the wild-type *Sye*7942 results in notable alterations in cellular gene expression [19]. To comprehensively investigate the role of the C252Y mutation in HLHT adaptation, global RNA-sequencing was carried out to analyze the transcriptional variances between the wild-type and C252Y mutant cultivated under both NTNL and HLHT conditions.

Our results revealed that there were 46.2% (1247 genes) and 27.8% (751 genes) of the genes that exhibited differential expression between the wild-type and mutant strain under NTNL and HLHT conditions, respectively (Supplemental Figures S12 and S13, Datasets S1 and S2). This indicates a wide-ranging impact of the mutation on gene regulation in *Sye*7942. For the following further analysis, we selected genes with a transcription fold change log2 > 0.5 and *p* < 0.05 from the datasets (Datasets S1 and S2).

Specifically, the mutant demonstrated enhanced nutrient uptake, with the potassium, sulfate, phosphate, carbon (including CO_2_ and bicarbonate uptake genes), and nitrate transporters being the most highly expressed compared to the wild-type strain under NTNL conditions (Dataset S1: nutrient transporters). Furthermore, numerous genes involved in photosynthesis, respiration, and the Calvin–Benson– Bassham (CBB) cycle consistently showed higher expression in the mutant (Dataset S1). Interestingly, genes encoding mitochondrial-type cytochrome c oxidase were downregulated in the mutant compared to the wild-type, as this enzyme is important for dark respiration metabolism in cyanobacteria [21,22,23]. Moreover, the mutant strain displayed increased expression of genes encoding ribosomal proteins, indicating a more efficient translation process. Additionally, genes involved in glycogen, fatty acid, chlorophyll a (*Chl* a), and carotenoid synthesis were also upregulated in the mutant strain (Dataset S1). Those could benefit more efficient photosynthetic carbon fixation and accelerate cell division, thus contributing to a notably higher autotrophic growth rate in the C252Y mutant compared to the wild-type strain under NTNL conditions (Figure 1A).

**Figure 4:**
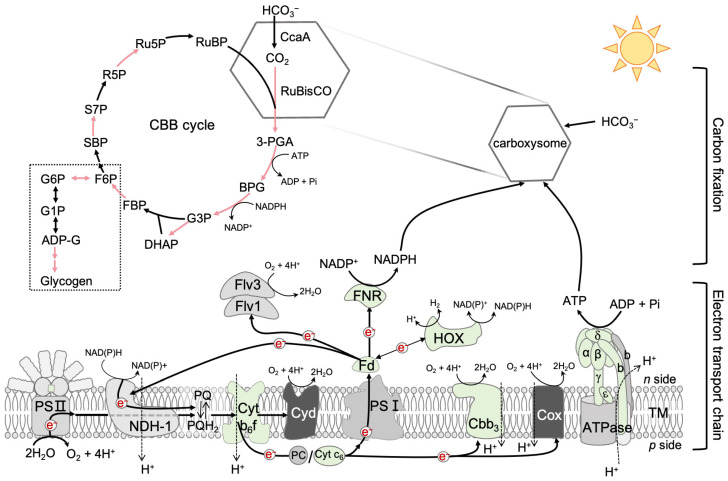
Alterations in gene expression of the electron transport chain and carbon fixation genes resulting from the C252Y mutation under HLHT conditions (40 °C, 2000 μmol photons m^−2^ s^−1^). Genes with a transcription fold change log2 > 0.5 and *p* < 0.05 were included in the analysis. Protein complexes encoded by multiple genes were indicated as up- or downregulated only if these genes exhibited consistent up- or downregulation. Otherwise, the expression levels of the protein complexes were consistent between the wild-type and C252Y mutant strains. In the electron transport chain, upregulated protein complexes were depicted in green color, downregulated complexes in dark gray, and consistent complexes in light gray. For carbon fixation, upregulated enzymes were represented as red arrows and the consistent complexes as black arrows. No genes related to carbon fixation showed downregulation in the C252Y mutant compared to the wild-type strain under HLHT conditions. Abbreviations: TM, thylakoid membrane; ATPase, ATP synthase; Cox, mitochondrial-type Cyt c oxidase; Cbb3, Cbb3-type cytochrome c oxidase; Cyd, Cyt bd quinol oxidase; Cyt, cytochrome; PC, plastocyanin; Fd, ferredoxin; Flv, flavodiiron protein heterodimer; FNR, Fd-NADP^+^ oxidoreductase; Hyd, bidirectional hydrogenase; NDH, NAD(P)H dehydrogenase; PS, photosystem; PQ, plastoquinone; PQH_2_, plastoquinol; CBB cycle, Calvin–Benson– Bassham cycle; RuBP, ribulose 1,5-bisphosphate; 3-PGA, 3-phosphoglycerate; BPG, 1,3-bisphospho- glycerate; G3P, glyceraldehyde 3-phosphate; DHAP, dihydroxyacetone phosphate; FBP, fructose 1,6-bisphosphate; F6P, fructose 6-phosphate; SBP, sedoheptulose 1,7-bisphosphate; S7P, sedoheptulose 7-phosphate; R5P, ribose 5-phosphate; Ru5P, ribulose 5-phosphate; Rubisco, RuBP carboxylase/oxygenase; G6P, glucose 6-phosphate; G1P, glucose 1-phosphate; ADP-G, ADP-glucose; and CcaA, carbonic anhydrase.

Under HLHT conditions, the gene expression patterns differed between the wild-type and C252Y mutant strains (Supplemental Figure S12B, Dataset S2). Genes encoding potassium, sulfate, and nitrogen transporters were also upregulated in the mutant compared to the wild-type strain (Dataset S2: nutrient transporters). Although most carbon uptake genes showed no significant changes between the wild-type and C252Y mutant strains, sodium-dependent bicarbonate transporter and iron transporter genes were downregulated in the mutant compared to the wild-type strain. Furthermore, the expression of genes related to the electron transport chain, carbon metabolism, and transcription regulators exhibited distinct alterations between the wild-type and C252Y mutant strains under HLHT conditions (Table 1, Figure 4), suggesting a unique response to HLHT in the C252Y mutant compared to the wild-type strain.

**Figure 5:**
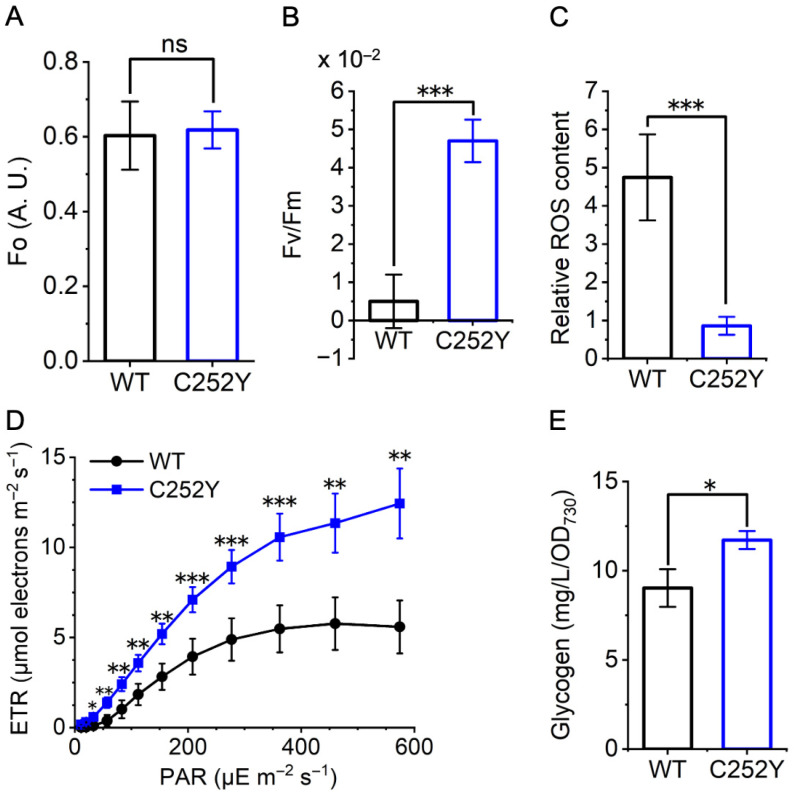
The effects of the C252Y mutation on photosynthesis, ROS generation, and intracellular glycogen content in *Sye*7942 under HLHT conditions (40 °C, 2000 μmol photons m^−2^ s^−1^). (**A**) Minimal fluorescence yield (Fo). (**B**) Variable fluorescence to maximum fluorescence (Fv/Fm). (**C**) Intracellular ROS level. (**D**) Electron transport rate (ETR). (**E**) Intracellular glycogen content. One star, significant differences (*p* < 0.05); two stars, highly significant (*p* < 0.01); three stars, extremely significant (*p* < 0.001); and ns, no significant difference (n > 3). WT, wild-type *Sye*7942; C252Y, the C252Y mutant. ROS, reactive oxygen species; PAR, photosynthetically active radiation.

In contrast to the widespread upregulation of genes encoding protein complexes in the electron transport chain observed in the mutant under NTNL conditions (Dataset S1), only specific genes within this category showed increased transcript levels in the mutant compared to the wild-type under HLHT conditions (Table 1, Figure 4). Genes involved in photosynthesis, such as those encoding phycobilisome linker polypeptide (ApcC, *apcC*), cytochrome b_6_f complex (Cyt b_6_f, *petA* and *petD*), c-type cytochrome (Cyt c_6_), ferredoxin (Fd, *petF1*), and ferredoxin-NADP^+^ reductase (FNR, *petH*), were upregulated in the mutant (Table 1, Figure 4). In the C252Y mutant, the transcription of two photosystem I (PSI) reaction center subunits, namely subunit IV and PsaB, was downregulated, while another subunit, PsaK, showed an upregulation compared to the wild-type strain. The C252Y mutation likely reshaped the photosynthetic chain in *Sye7942* to improve solar energy utilization and photosynthetic efficiency under HLHT conditions. To verify this hypothesis, the photosynthetic parameters were detected using a DUAL-PAM-100 fluorometer. The Fo values were comparable between the wild-type and mutant strains (Figure 5A), but the Fv/Fm values, negligible in the wild-type strain, were significantly enhanced in the mutant (Figure 5B). These results suggest a more active PSII in the mutant compared with the wild-type strain under HLHT conditions. Furthermore, the photosynthetic electron transport rate was significantly higher in the mutant than in the wild-type strain (Figure 5D). While photoinhibition was evident in the wild-type strain as light intensity rose, the mutant showed no signs of photoinhibition even under intense illumination (Figure 5D). This indicates reduced photoinhibition in the mutant under HLHT conditions, which is consistent with decreased ROS levels in the mutant cells (Figure 5C). In *Sye*7942, there are three types of cytochrome terminal oxidase. The genes encoding two of them, cytochrome c oxidase and cytochrome ubiquinol oxidase, were consistently downregulated, while the upregulation of the Cbb3 (cbb3-type cytochrome c oxidase) was observed (Table 1, Figure 4). Notably, the expression levels of the genes encoding all components of the F_1_-subcomplex and the b’ subunit of the F_o_-subcomplex were upregulated in the mutant compared to the wild-type (Table 1, Figure 4), consistent with the protein levels illustrated in Figure 2. Additionally, the genes encoding hydrogenase, vital for cyanobacterial growth to mitigate photoinhibition under high light stress conditions [24], were also upregulated in the C252Y mutant compared to the wild-type strain. These results suggest that the C252Y mutation enhances the electron transport chain, suggesting improved solar energy conversion efficiency.

The CBB cycle uses NADPH and ATP from the light reaction to convert CO_2_ into carbohydrates, serving as the main energy and electron sink. This process is the primary pathway of photosynthetic carbon fixation and greatly impacts the tolerance of photosynthetic organisms to abiotic stresses [25,26]. Therefore, differences in gene transcription related to carbon fixation were evaluated between the wild-type and mutant strains under HLHT conditions. As shown in Figure 4, the majority of the genes associated with the CBB cycle were upregulated in the mutant compared with the wild-type strain. Specifically, the gene (*SYNPCC7942_RS07280*) responsible for the small subunit of ribulose bisphosphate carboxylase (RuBisCO), exhibited a 2.4-fold upregulation (*p* = 0.00027), and the gene (*SYNPCC7942_RS11870*) encoding fructose-1,6-bisphosphatase (FBPase)/sedoheptulose-1,7-bisphosphatase (SBPase), which catalyze the hydrolysis of fructose-1,6-bisphosphate and sedoheptulose-1,7-bisphosphate [27], showed a 2.3-fold increase (*p* = 0.000000016) in expression in the mutant relative to the wild-type strain under HLHT conditions (Table 1, Dataset S2). Genes involved in the oxidative phase of the pentose phosphate (OPP) pathway, which produce NADPH and support carbon fixation, were also upregulated (Supplemental Figure S14, Table S2). Additionally, three crucial genes associated with glycogen synthesis were upregulated in the mutant compared to the wild-type strain: *SYNPCC7942_RS11870* (encoding Glucose-6-phosphate isomerase; 1.6-fold, *p* = 0.0054), *glgA* (2.0-fold; *p* = 0.012), and *glgB* (1.5-fold; *p* = 0.00000026) (Table 1). These changes align with the observed higher cellular glycogen content in the mutant, as illustrated in Figure 5E. Furthermore, beyond carbon fixation, genes associated with protein synthesis, including those encoding ribosomes and amino acid-tRNA ligases, as well as those responsible for fatty acid synthesis, were upregulated in the mutant compared to the wild-type under HLHT conditions (Dataset S2).

The findings suggest that the C252Y mutation in *Sye*7942 caused significant and widespread changes in gene expression under HLHT conditions. To further understand its implications, we investigated the impact of this mutation on the transcription of genes involved in vital regulatory systems for cellular adaptation to HLHT conditions. The majority of the highlight-responsive regulators, including *ntcA*, *nusG*, *sigA2*, *rpoB*, *SYNPCC7942_RS03440*, *SYNPCC7942_RS07940*, *SYNPCC7942_RS02895*, and *ssrA* [28,29,30,31], showed no significant differences between the wild-type and mutant strains (Dataset S2). However, two *sigF* genes (*SYNPCC7942_RS07720* and *SYNPCC7942_RS09065*) were downregulated by 1.7-fold (*p* = 0.000004) and 2.3-fold (*p* = 0.000009), respectively, in the mutant compared with the wild-type (Table 1). The two other sigma factors encoding genes, *sigC* and *rpoD*, were upregulated by 3.6 (*p* < 0.01) and 1.5-fold (*p* = 0.0039), respectively, in the mutant (Table 1, Dataset S2). Additionally, there was a 1.7-fold (*p* = 0.0049) increase in the expression of the gene encoding the heat-inducible transcriptional repressor, HrcA, in the mutant compared to the wild-type strain (*p* = 0.0049). Genes encoding heat shock proteins, *danK*, *groEL*, *clpB*, and *htpG*, were downregulated by 1.8 (*p* = 0.011), 1.8 (*p* = 0.007), 2.3 (*p* = 0.000041), and 3.6-fold (*p* < 0.01) in the mutant compared to the wild-type (Table 1, Dataset S2). In the mutant strain, the genes responsible for circadian transcriptional regulators, *rpaA* and *rpaB*, showed a decrease in expression by 1.9 and 2.2-fold, respectively, when compared to the wild-type under HLHT conditions (Table 1, Dataset S2). The *rpaA* and *rpaB* have been observed to significantly contribute to the high growth rate and HLHT tolerance of cyanobacteria [19,32,33,34]. These results indicate that the C252Y mutation comprehensively rewrote the transcriptional regulation hierarchy in *Sye*7942 under HLHT conditions.

## 3. Discussion

In cyanobacteria, ATP synthase utilizes the transmembrane PMF generated through photosynthesis and respiration to convert ADP and Pi into ATP, the primary energy source for the cells [10]. This process, depicted in Figure 4, is fundamental for ATP synthesis. A specific point mutation, F_1_-α-C252Y, in ATP synthase can allow *Sye*7942 to exhibit the fast-growing phenotype of the strain *Synechococcus* UTEX 2973 under HLHT conditions, resulting in a doubling time as short as 1.9 h [9,19,35]. This mutation boosts the cellular activity of ATP synthase, which was hypothesized to contribute to the improved rapid growth and adaptability of the mutant strain under short-term (2 to 4 h) HLHT conditions by increasing ATP production for repairing damage [9]. It is worth noting that the mechanism by which photosynthetic organisms respond to short-term (up to 6 h) stresses differs from their response to long-term (days) stresses [36]. And the exact mechanisms through which the mutation impacts the cellular activity of ATP synthase are still unclear.

Hence, we conducted a comparative analysis to examine the mutation’s impact on ATP synthase activity, as well as the abundance of ATP synthase and its subunits within the mutant and wild-type strains under both NTNL and constant HLHT conditions. The C252Y mutation led to higher cellular levels of ATP synthase compared to the wild-type, particularly under HLHT conditions (Figure 3C). It is noteworthy that both strains exhibited a substantial presence of the F_1_ subcomplex under NTNL conditions, which significantly decreased under HLHT conditions (Figure 3C), indicating the F_1_ subcomplex’s instability under HLHT conditions. Additionally, no other subcomplexes were observed under either NTNL or HLHT conditions, implying the efficient assembly of the F_1_ subcomplex in cyanobacteria. Furthermore, the content of most subunits of the F_1_ subcomplex, except for the AtpB, decreased in the wild-type strain under HLHT conditions compared to NTNL conditions, whereas all subunits increased in the mutant strain under HLHT conditions (Figure 2A). In contrast, the levels of all subunits within the F_o_ subcomplex remained relatively consistent in both the mutant and wild-type strains under both NTNL and HLHT conditions, and were even higher under HLHT conditions, except for the b subunits (Figure 2B). These results suggest the instability of the F_1_ subcomplex under both NTNL and HLHT conditions, contrasting with the relative stability of the F_o_ subcomplex even under HLHT conditions. This implies that the F_1_ subcomplex may play a vital role in controlling ATP synthase complex levels, and the b subunit likely plays a significant role in the assembly or dissociation of F_1_ subcomplex into or from F_o_ subcomplex in cyanobacteria. The elevated AtpA and AtpB levels in the mutant (refer to Figure 2), likely due to higher expression (refer to Table 1, Figure 4, and Dataset S2), suggest that the mutation may have enhanced the cellular ATP synthase complex content (Figure 3C) by boosting AtpA and AtpB protein levels. Research has shown that the crucial initial step in ATP synthase assembly is the dimerization of the AtpA and AtpB subunits [10]. Therefore, higher AtpA and AtpB levels could facilitate ATP synthase assembly and elevate the ATP synthase levels. In addition to affecting AtpA and AtpB, the C252Y mutation positively influenced the transcription levels of unstable ATP synthase subunits, including the b subunit of the F_o_ subcomplex and the other subunits of the F_1_ subcomplex, relative to the wild-type under HLHT conditions (refer to Table 1, Figure 4, and Dataset S2). This probably led to increased cellular content of these subunits in the mutant (Figure 2), contributing to the higher ATP synthase levels in the mutant strain (Figure 3C). Further study is needed to explore if the mutation affects the stability of AtpA, ATP synthase subcomplexes, and ATP synthase. These findings suggest that AtpA plays a crucial role in regulating the intracellular levels of ATP synthase at various levels, with the mutation acting as a critical site for its overall function within ATP synthase in cyanobacteria.

The higher levels of intracellular ATP synthase (Figure 3C) and consistent ATP synthesis activity between the wild-type and mutant ATP synthase (Figure 3B) suggest enhanced intracellular ATP synthesis activity in the mutant compared to the wild-type. Furthermore, the significantly improved photosynthetic efficiency (Figure 5) in the mutant is expected to generate a higher PMF, powering ATP synthase rotation and ATP generation. These findings anticipate that the mutant would demonstrate elevated ATP productivity under HLHT conditions, consistent with previous reports [9]. The heightened ATP production is recognized to contribute to the rapid growth and stress tolerance of cyanobacteria under HLHT conditions [9]. Interestingly, the intracellular ATP levels were found to be similar to those of the wild-type strain (Figure 3D). One possible explanation for this finding could be the stimulation of increased metabolism in the mutant under HLHT conditions, including a faster growth rate (Figure 1), enhanced carbon fixation efficiency (Figure 5E), and improved stress adaptation (Figure 1 and Figure 5). These boosted metabolic activities, fueled by ATP, suggest an elevated intracellular ATP consumption that potentially counteracts the higher intracellular ATP synthesis rate in the mutant. This delicate balance could help maintain a stable ATP concentration in vivo.

The NADPH and ATP generated during photosynthesis in cyanobacteria play a crucial role in aiding carbon fixation. However, if the influx of photons surpasses the conversion capacity of the photosystem or the electron and energy sink capability of the CBB cycle, the electron transport chain becomes overly reduced, leading to photoinhibition and a significant decline in photosynthetic efficiency [37]. The photosystem and CBB cycle are highly sensitive to HLHT conditions [3,38,39]. Fine-tuning the photosystem and improving the CBB cycle activity are effective strategies for boosting the tolerance of photosynthetic organisms to HLHT [26,40,41]. To investigate how mutations affect *Sye*7942’s adaptation to HLHT, we compared differences in the photosystem and CBB cycle between the wild-type and C252Y mutant under HLHT conditions.

In the photosystem, the mutant showed overexpression of genes encoding ApcC, Cyt b_6_f, Cyt c_6_, Fd, and FNR under HLHT conditions compared to the wild-type (Figure 4, Table 1). ApcC, an important APC (allophycocyanin) linker protein, enhances APC complex stability [42,43,44], facilitating efficient solar energy transfer from light-harvesting antennas to reaction centers. This increase in *apcC* expression would enhance solar energy utilization in the mutant under HLHT conditions. Cyt b_6_f is a central component in the photosynthetic electron transport chain, with the oxidation of reduced PQ by Cyt b_6_f considered a rate-limiting step in cyanobacteria and higher plants [45,46,47]. Studies have shown that increasing the expression of Cyt b_6_f or Cyt c_6_ could enhance electron flow to PSI, thereby boosting the photosynthetic electron transport rate, overall photosynthetic efficiency, and adaptability to high-light conditions [48,49,50,51]. Fd acts as a crucial electron acceptor from PSI, transferring electrons to FNR, the final electron acceptor that converts NADP^+^ into NADPH (Figure 4). Co-overexpressing Fd and FNR boosts NADPH generation, prevents photodamage to PSI caused by over-reduction, decreases ROS production, enhances photosynthetic efficiency, and improves stress adaptability [52,53]. Furthermore, genes encoding Cbb3 and Hyd complexes (bidirectional hydrogenase) were also upregulated in the mutant compared with the wild-type strain under HLHT conditions. While the function of Cbb3 genes in cyanobacteria remains unclear, they are typically expressed only under microaerobic conditions in many bacteria [54]. Hyd complexes are essential for high-light stress adaptation in cyanobacteria [55,56]. These results indicate that the mutation resulted in an optimized electron transport chain, improving solar energy conversion efficiency, decreasing photoinhibition, and restricting ROS production under HLHT conditions, as shown in Figure 5.

The CBB cycle, the initial pathway for assimilation of CO_2_, comprises 11 enzymes arranged into three stages: carboxylation, reduction, and RuBP regeneration (Figure 4) [57]. The critical rate-limiting steps in the cycle are carboxylation and RuBP regeneration, contributing to inefficient carbon fixation [58,59]. These processes are mainly regulated by three key enzymes—Rubisco for carboxylation, and FBPase/SBPase for RuBP regeneration [27,58,60,61]. In cyanobacteria, the unique dual enzyme FBPase/SBPase processes both FBPase and SBPase activities [27,62,63,64]. In the C252Y mutant, six out of 11 enzyme-encoding genes were upregulated compared to the wild-type strain under HLHT conditions (Figure 4, Table 1). Specifically, the genes encoding FBPase/SBPase and the small subunit of Rubisco were upregulated, which would contribute to the fast growth rate and HLHT-stress adaptability. Studies in higher plants, algae, and cyanobacteria have shown that overexpressing FBPase/SBPase significantly enhances carbon fixation, leading to increased growth rate, photosynthetic efficiency, and stress resilience [61,65,66,67,68]. The active sites of Rubisco are located on the large subunits, while the small subunit plays a crucial role in Rubisco stability [58]. Regulation of the small subunit expression affects Rubisco abundance, thereby influencing overall Rubisco activity. Increasing the expression of the small subunit in the C252Y mutant could enhance the intracellular activity of Rubisco, thereby improving the carbon fixation efficiency of the mutant under HLHT-stress conditions. Moreover, the genes involved in catalyzing the OPP pathway were found to be upregulated in the mutant compared to the wild-type (Supplemental Figure S14, Table S2). The OPP pathway generates vital reductive power, NADPH, and a precursor source for the CBB cycle [69,70]. Modulating the expression of these enzymes could enhance key CBB cycle enzyme functions, ultimately optimizing carbon fixation. Glycogen serves as a major carbon storage molecule in cyanobacteria, synthesized by enzymes, including ADP-glucose pyrophosphorylase (G1P adenylyl transferase), glycogen synthases (GlgA), and 1,4-α-glucan branching enzyme (GlgB) [71,72]. Mutants lacking these enzymes are vulnerable to stress, such as intense light exposure [40,73]. In the C252Y mutant, the *glgA* and *glgB* were upregulated compared with the wild-type strain under HLHT conditions (Table 1, Figure 4). The gene *SYNPCC7942_RS10295*, which encodes glucose-6-phosphate isomerase, was also upregulated in the mutant. This enzyme facilitates the conversion of fructose-6-phosphate (F6P) to glucose-6-phosphate (G6P), a precursor for glycogen synthesis. The strain with its overexpression shows increased tolerance to high light intensity [74]. In line with these results, the mutant exhibited a notable increase in intracellular glycogen levels compared to the wild-type strain, as shown in Figure 5E. These results indicate that the genetic transcription changes in the C252Y mutant have boosted its ability to store energy, thereby improving its capacity for photosynthetic carbon fixation. As a result, the mutant was able to substantially enhance its growth rate even when exposed to constant HLHT conditions (Figure 1).

The C252Y mutation in the AtpA subunit not only altered gene transcription levels related to ATP synthase, photosystem, and carbon fixation, but also greatly influenced various cellular processes such as protein synthesis, nutrient absorption, and lipid production (Dateset S1 and S2). This overall transcriptome restructuring in the mutant might be a result of regulatory system changes. Surprisingly, the levels of high-light transcription regulators such as NtcA, FurA, IdiB, Rre1, NblS, SsrA, and NblR were consistent between the wild-type and mutant strains under HLHT conditions. In comparison to the wild-type, the mutant displayed downregulation of the high-light negative responsive sigma factors, two SigF factors [75], and the upregulation of the sigma factor SigC in the mutant (Dataset S2), which is crucial for high-temperature acclimation in cyanobacteria [76]. This may explain the increased expression of genes aiding in adaptation to HLHT. Additionally, the repressor HrcA, responsible for inhibiting the expression of heat shock proteins (HSPs) [77], was upregulated in the mutant. This upregulation aligned with reduced expression of HSP-encoding genes, including DnaK, GroEL, ClpB, and HtpG (Table 1). HSPs are critical for safeguarding cells during thermal stress by stabilizing protein structures and refolding misfolded polypeptides, making their upregulation essential at high temperatures [78]. The decreased HSPs expression in the mutant suggests lower thermal damage compared to the wild-type under HLHT conditions. Furthermore, the global circadian regulators RpaA and RpaB were downregulated in the mutant compared to the wild-type. These two circadian regulators individually bind to the promoters of over 150 genes, including sigma factors [30,34,79,80]. The downregulation of RpaA was reported to be associated with the rapid growth of cyanobacteria [19,32]. RpaB is crucial for the high-light adaptation of cyanobacteria, with its functional regulation dependent on phosphorylation levels, while its protein content and transcription levels remain consistent under varying light conditions [34,81]. The impact of RpaB downregulation in cyanobacteria is currently not fully understood. The downregulation of circadian regulators in the mutant possibly influenced the overall cellular transcriptome, facilitating the rapid growth and efficient photosynthetic carbon fixation of the C252Y mutant under HLHT conditions.

This study delved into the specific roles of the C252Y mutation in the AtpA subunit of ATP synthase, focusing on its role in regulating intracellular energy supply and metabolic processes to enhance the HLHT tolerance of *Synechococcus*. The results indicate that increasing ATP production and metabolic remodeling via the mutation are critical strategies for *Synechococcus* to survive in HLHT. The conservation of the 252nd amino acid as tyrosine in the AtpA subunit of cyanobacteria suggests that the mechanism utilized in the C252Y mutant may have widespread applicability among cyanobacteria for adapting to challenging environments throughout their evolutionary history and a variety of habitats on Earth [9]. The study highlights the significance of comprehensive regulation in boosting the abiotic stress tolerance of *Synechococcus*, with the AtpA subunit playing a crucial role in this process. These findings could be pivotal in the development of genetically modified photosynthetic organisms with enhanced resilience to stress and improved efficiency in photosynthesis.

## 4. Materials and Methods

### 4.1. Strains and Culture Conditions

The wild-type *Sye*7942 used in this study was obtained from Xudong Xu’s lab at the Institute of Hydrobiology, Chinese Academy of Sciences. The C252Y mutant was constructed from the wild-type in our previous study [9]. The *Sye*7942 strains were maintained on BG11 agar plates at 30 °C with 50 μmol photons m^−2^ s^−1^ light illumination (warm white) and were routinely grown with shaking in liquid BG11 medium under the same conditions. For determining parameters, the cultures were inoculated into column photobioreactors containing 60 mL BG11 medium with an initial OD_730_ of 0.02, and were grown in a MC-1000 muti-cultivator bioreactor (Photon Systems Instruments, Drásov, Czech Republic) under NLNT (30 °C, 280 μmol photons m^−2^ s^−1^; cool white), 42 °C, 2000 μmol photons m^−2^ s^−1^ (cool white), HLHT (40 °C, 2000 μmol photons m^−2^ s^−1^; cool white), 41 °C, 1500 μmol photons m^−2^ s^−1^ (cool white), and 41 °C, 2000 μmol photons m^−2^ s^−1^ (cool white) conditions respectively. All the cultures were bubbled with 3% CO_2_, and growth was monitored by measuring the optical density at 730 nm (OD_730_).

The *E. coli* strain DK8 (*HfrPO1 bglR thi1 relA1 ilv::Tn10 ΔatpBEFHAGDC*) ilv::Tn10, tetracycline resistant) [82] was obtained from Yirong Sun at Guangzhou Institutes of Biomedicine and Health, Chinese Academy of Sciences. In this strain, the genes for ATP synthase were completely deleted.

### 4.2. Plasmid and Strain Construction

The two operons that encode subunits of ATP synthase were cloned into the pETDuet-1 plasmid, where they were expressed by two T7 promoters and included a 6xHis tag at the N-terminus of the β subunit. DNA fragments of the two operons were amplified from the wild-type *Sye*7942 genome, and pETDuet-1 was linearized by PCR amplification using a high-fidelity DNA polymerase (P520, Vazyme Biotech Co., Ltd. Nanjing, China). Plasmid construction was carried out via Gibson Assembly using the ClonExpress^®^ Ultra One Step Cloning Kit (C115, Vazyme Biotech Co., Ltd.). The resulting plasmid, named pWL8, was introduced into *E. coli* DK8 strain to express wild-type ATP synthase from *Sye*7942. The plasmid (pWL25) expressing the C252Y mutation in ATP synthase was constructed using pWL8 as a template. The primers utilized in this study are listed in Supplemental Table S3.

### 4.3. Immunoblot Analysis

Polyclonal antibodies for the subunits of ATP synthase were produced by GenScript (Nanjing, China). Total protein was extracted from the wild-type *Sye*7942 and C252Y mutant strains. The protein samples were standardized based on chlorophyll quantity (containing 1.6 μg *Chl* a) and verified through stained SDS-PAGE (see Supplemental Figure S2). Equal amounts of protein were resolved by SDS-PAGE or native PAGE using a 4–20% gradient polyacrylamide gel (P0469S, Beyotime Biotechnology, Shanghai, China). The proteins were then blotted onto nitrocellulose membranes and detected using the corresponding antibodies. The densitometric quantification of these results is available in Supplemental Table S1.

### 4.4. Protein Purification

*E. coli* strain DK8, with the plasmids pWL8 and pWL25, was used to prepare the wild-type and the C252Y mutant ATP synthase. The overnight cultures were inoculated into 2 L of LB medium at a ratio of 1:100 and grown at 37 °C with shaking for about 4 h in the presence of 50 μg/mL ampicillin. Following this, 1 mM IPTG was introduced into the cultures, which were further incubated at 25 °C with shaking for an additional 4 h. Cells were collected and resuspended in an ice-cold buffer containing 50 mM Tris/base (pH 8.0), 200 mM NaCl, and 0.2 mM DTT. All the subsequent steps were performed at 4 °C. Cells were broken by passing through a French Press (twice, at 10,000 psi) in the presence of 1× protein inhibitor (P1006; Beyotime Biotechnology). Unbroken cells were discarded after centrifuging at 8000 g for 10 min. The resulting supernatant was then centrifuged at 20,000 g for 1 h to collect the membrane vesicles. The pellet was resuspended in an ice-cold extraction buffer containing 50 mM Tris/base (pH 7.5), 100 mM KCl, 250 mM sucrose, 5 mM MgCl_2_, 0.1 mM EDTA, 0.2 mM DTT, 0.5% NaCl, 2.5% glycerol, 20 mM imidazole, 0.05% n-Dodecyl-β-D-maltoside, and 1× protein inhibitor (P1006, Beyotime Biotechnology), and incubated for 2 h on a shaking platform. The resulting supernatant was centrifuged at 20,000 g for 3 h. The ATP synthase complex was isolated from the obtained supernatant utilizing HisTrap HP affinity chromatography.

### 4.5. Liposome Preparation and ATP Synthase Activity Assessment

Liposome and proteoliposome preparations were carried out in accordance with the reported protocol [83]. The activity of ATP synthase was assessed using the acid/base transition method detailed in our prior study [9].

### 4.6. Determination of Intracellular ATP Content

Intracellular ATP levels were measured and quantified using an ATP bioluminescence assay kit (S0026; Beyotime Biotechnology) following the instructions.

### 4.7. RNA-Seq Analysis

RNA sequencing was performed by Allwegene Technology (Tianjin, China). RNA was extracted using the TRIzol method (Invitrogen, Carlsbad, CA, USA) and treated with RNase-free DNase I (Takara, Kusatsu, Japan). The quality of the RNA was evaluated using Agilent 2100 Bioanalyzer (Agilent Technologies, Santa Clara, CA, USA), as well as the NanoDrop spectrophotometer (Thermo Scientific, Wilmington, DE, USA), and confirmed on 1% agarose gels. Each RNA sample used 3 μg for preparation, with rRNA depletion carried out using the Vazyme Ribo-off rRNA depletion kit (bacteria) (Vazyme Biotech Co., Ltd., Nanjing, China). Libraries for sequencing were constructed using the NEBNext UltraTM RNA library Prep Kit (New England Biolabs, Inc., Ipswich, MA, USA). Library quality was assessed on the Agilent Bioanalyzer 2100 system. The library preparations were sequenced on an Illumina Novaseq 6000 platform by Beijing Allwegene Technology Company Limited (Beijing, China), and paired-end 150 bp reads were generated. Raw data (raw reads) of fastq format were first processed through in-house Perl scripts to obtain high-quality and clean data. The raw reads were aligned to the reference genome sequence retrieved from the National Center for Biotechnology Information (NCBI) BioProject database (accession number: PRJNA1069991) using Bowtie2 v2.2.6. HTSeq v 0.5.4 p3 was used to count the number of reads mapped to each gene. The sequencing quality details are provided in Supplemental Table S4. Gene expression levels were estimated by fragments per kilobase of transcript per million fragments mapped (FPKM). Differential expression analysis of two conditions/groups (four biological replicates per condition/group) was performed using the DESeq R package (1.10.1). The resulting *p*-values were adjusted using Benjamini and Hochberg’s approach for controlling the false discovery rate. Genes with an adjusted *p*-value < 0.05 found by DESeq were assigned as differentially expressed. The principal component analysis is available in Supplemental Figure S13.

### 4.8. Assessing Intracellular ROS Levels

Intracellular ROS levels were assessed using a Reactive Oxygen Species Assay Kit (S0033S, Beyotime Biotechnology) following the manufacturer’s guidelines.

### 4.9. Photosynthetic Parameter Determination

Rapid light response curves, Fo, and Fv/Fm measurements were assessed using a DUAL-PAM-100 fluorometer (Heinz Walz GmbH, Nuremberg, Germany) after 15 min of dark adaptation. The minimal fluorescence after dark adaptation (Fo) was measured under measuring light (5 µE m^−2^ s^−1^), while the maximum fluorescence (Fm) was determined after a saturating pulse (200 ms, 7044 µE m^−2^ s^−1^). Rapid light response curves were generated using actinic light intensities ranging from 11 to 574 µE m^−2^ s^−1^. The fluorescence yields F’ and Fm’ were recorded before and after the final saturating light pulse, respectively, at the end of each actinic illumination period. The electron transport rate (ETR) was calculated using the DUAL-PAM software (Dual PAM v1.19 software) based on the following formula:

ETR = (Fm’ − F’)/Fm’ × PAR × 0.84 × 0.5

PAR (photosynthetically active radiation) is the actinic light intensity.

### 4.10. Intracellular Glycogen Content Measurement

The detection of intracellular glycogen was conducted following the methods outlined in our previous research [9].

## 5. Conclusions

This study revealed that the C252Y mutation not only boosted ATP synthesis activity through increased ATP synthase protein levels but also altered the cellular transcriptome, leading to enhanced photosynthetic activity, carbon fixation, faster growth, and improved tolerance to HLHT in *Sye*7942. These discoveries would be beneficial for the future optimization of photosynthetic organisms. However, more research is needed to understand the specific mechanisms through which the C252Y mutation influences ATP synthase content, whether by stabilizing the complex or regulating transcription. Additionally, its effects on the cellular transcriptome, such as through increased ATP production, interactions with transcription regulators, or the potential regulatory role of AtpA, require further in-depth research.

## Figures and Tables

**Table 1:** Gene expression changes in the C252Y mutant compared to the wild-type under HLHT (40 °C, 2000 μmol photons m^−2^ s^−1^) conditions. Genes related to photosynthesis, ATP synthase, respiration, the Calvin–Benson– Bassham cycle, glycogen synthesis, sigma factors, transcriptional regulation, and heat shock proteins were presented here: fold change log2 > 0.5, *p* < 0.05.

Gene ID	Gene Functional Identification	Fold Change (Times)
**Photosynthesis**		
SYNPCC7942_RS01655	phycobilisome linker polypeptide; *apcC*	2.8
SYNPCC7942_RS05020	ferredoxin-NADP^+^ reductase; *petH*	2.3
SYNPCC7942_RS06310	apocytochrome f; *petA*	2.6
SYNPCC7942_RS11855	cytochrome b_6_-f complex subunit IV; *petD*	1.6
SYNPCC7942_RS07660	Ferredoxin; *petF1*	3.3
SYNPCC7942_RS08315	c-type cytochrome	1.6
SYNPCC7942_RS04735	photosystem I reaction center subunit; *psaK*	1.7
SYNPCC7942_RS06760	photosystem I reaction center subunit IV	−1.4
SYNPCC7942_RS10390	photosystem I core protein; *psaB*	−1.7
**ATP synthase**		
SYNPCC7942_RS01700	F_o_F_1_ ATP synthase subunit B	2.0
SYNPCC7942_RS01705	ATP synthase F_1_ subunit delta;	2.0
SYNPCC7942_RS01710	F_o_F_1_ ATP synthase subunit alpha	2.0
SYNPCC7942_RS01715	F_o_F_1_ ATP synthase subunit gamma	1.7
SYNPCC7942_RS11770	F_o_F_1_ ATP synthase subunit beta	1.6
SYNPCC7942_RS11775	ATP synthase F_1_ subunit epsilon	1.9
**Respiratory**		
SYNPCC7942_RS10020	NAD(P)H-quinone oxidoreductase subunit D1; ndhD1	−1.8
SYNPCC7942_RS10025	NAD(P)H-quinone oxidoreductase subunit 5 (F1)	1.6
SYNPCC7942_RS11345	NAD(P)H-quinone oxidoreductase subunit N	2.2
SYNPCC7942_RS12620	NAD(P)H-quinone oxidoreductase subunit O	−1.8
SYNPCC7942_RS08980	cytochrome ubiquinol oxidase subunit I	−1.8
SYNPCC7942_RS01020	cbb3-type cytochrome c oxidase subunit II	2.2
SYNPCC7942_RS01025	cbb3-type cytochrome c oxidase subunit I	2.0
SYNPCC7942_RS12995	bidirectional hydrogenase complex protein; *hoxU*	2.8
SYNPCC7942_RS01415	bidirectional hydrogenase complex protein; *hoxE*	2.0
SYNPCC7942_RS13255	cytochrome c oxidase subunit II	−1.6
SYNPCC7942_RS03290	fumarate reductase/succinate dehydrogenase flavoprotein subunit	1.9
SYNPCC7942_RS07835	succinate dehydrogenase/fumarate reductase iron-sulfur subunit	−1.5
	**CBB cycle**	
SYNPCC7942_RS01250	type I glyceraldehyde-3-phosphate dehydrogenase; *gap*	2.0
SYNPCC7942_RS06460	triose-phosphate isomerase; *tpiA*	1.6
SYNPCC7942_RS05720	phosphoglycerate kinase	2.1
SYNPCC7942_RS08865	type I glyceraldehyde-3-phosphate dehydrogenase;	1.6
SYNPCC7942_RS07670	phosphoglycerate dehydrogenase; *serA*	1.4
SYNPCC7942_RS11870	class 1 fructose-bisphosphatase; *fbp*	2.3
SYNPCC7942_RS02970	ribose-5-phosphate isomerase; *rpiA*	2.6
SYNPCC7942_RS07280	ribulose bisphosphate carboxylase small subunit	2.4
**Glycogen synthesis**		
SYNPCC7942_RS10295	glucose-6-phosphate isomerase	1.6
SYNPCC7942_RS05570	alpha-glucan branching protein; *glgB*	1.5
SYNPCC7942_RS12785	glycogen synthase; *glgA*	2.0
	**Sigma factor**	
SYNPCC7942_RS09380	RNA polymerase sigma factor; *sigC*	3.6
SYNPCC7942_RS03325	RNA polymerase sigma factor; *rpoD*	1.5
SYNPCC7942_RS07720	RNA polymerase sigma factor; *sigF*	−1.7
SYNPCC7942_RS09065	RNA polymerase sigma factor; *sigF*	−2.3
	**Transcriptional regulator**	
SYNPCC7942_RS03160	heat-inducible transcriptional repressor; *hrcA*	1.7
SYNPCC7942_RS07435	response regulator transcription factor; *rpaB*	−1.9
SYNPCC7942_RS00480	response regulator transcription factor; *rpaA*	−2.2
	**Heat shock protein**	
SYNPCC7942_RS12545	molecular chaperone; *dnaK*	−1.8
SYNPCC7942_RS03510	Chaperonin; *groEL*	−1.8
SYNPCC7942_RS05590	ATP-dependent chaperone; *clpB*	−2.3
SYNPCC7942_RS09205	molecular chaperone; *htpG*	−3.6

## Data Availability

The original contributions presented in this study are included in the article. Further inquiries can be directed to the corresponding author.

## Supplementary Materials

The following supporting information can be downloaded at: https://www.mdpi.com/article/10.3390/md24050152/s1. Figure S1: Growth of *Synechococcus elongatus* PCC 7942 (*Sye*7942) strains under various HLHT conditions; Figure S2: SDS-PAGE analysis of the total protein samples from *Sye*7942 strains grown under NTNL and HLHT conditions; Figure S3: The original figure of Figure 2, anti-α subunit; Figure S4: The original figure of Figure 2, anti-β subunit; Figure S5: The original figure of Figure 2, anti-γ and δ subunit; Figure S6: The original figure of Figure 2, anti-ε subunit; Figure S7: The original figure of Figure 2, anti-a subunit; Figure S8: The original figure of Figure 2, anti-b subunit; Figure S9: The original figure of Figure 2, anti-b’ subunit; Figure S10: The original figure of Figure 2, anti-c subunit; Figure S11: The original figure of Figure 3C, anti-γ subunit; Figure S12: Volcano plot comparing gene expression differences between the wild-type and C252Y mutant; Figure S13: Principal component analysis (PCA) of RNA sequencing data; Figure S14: Changes in gene expression of the oxidative phase of the pentose phosphate pathway (OPP) genes due to the C252Y mutation in HLHT conditions (the mutant vs. wild-type); Table S1: Qualification the Western blot data; Table S2: Differential gene expression analysis of the pentose phosphate pathways in the C252Y mutant versus the wild-type under HLHT conditions: fold change log2 > 0.5, *p* < 0.05; Table S3: Primers used in this study; Table S4: Sequencing data statistics. Datasets S1: Differences in transcriptomes between wild-type and C252Y mutant strains under NTNL conditions; Datasets S2: Differences in transcriptomes between wild-type and C252Y mutant strains under HLHT conditions.
