# Supplementary material for: Enhanced Synechococcus Growth Under Extended High-Light and High-Temperature Stress by the F1-α-C252Y Mutation in ATP Synthase: ATP Generation and Metabolic Network Remodeling
Source: Mar Drugs. 2026 Apr 25;24(5):152. doi: 10.3390/md24050152 (PMC13208690; doi:10.3390/md24050152)
Supplement: Supplementary file 1 [file marinedrugs-24-00152-s001.zip › marinedrugs-4223210-supplementary.pdf]

Enhanced *Synechococcus* Growth under Extended High Light and Temperature Stress by the F_1-α-C252Y Mutation in ATP Synthase: ATP Generation and Metabolic Network Remodeling

Linan Zhou [1,2,†], Wenjing Lou [2,3,4,†,*], Xin Guo [2,5], Siyan Yi [2,6], Wenhui Lou [7], Guodong Luan [2,3,4], and Xuefeng Lu [2,3,4,*]

1 College of Life Sciences, Qingdao Agricultural University, Qingdao 266109, China; zhouln@qibebt.ac.cn

2 Key Laboratory of Photoelectric Conversion and Utilization of Solar Energy, Qingdao Institute of Bioenergy and Bioprocess Technology, Chinese Academy of Sciences, Qingdao 266101, China; louwj@qibebt.ac.cn (W.L.); guoxin@qibebt.ac.cn (X.G.); CynthiaYisy@outlook.com (S.Y.); luangd@qibebt.ac.cn (G. L); lvxf@qibebt.ac.cn (X.L)

3 Shandong Energy Institute, Qingdao 266101, China

4 Qingdao New Energy Shandong Laboratory, Qingdao 266101, China

5 School of Life Sciences, Henan University, Kaifeng 475004, China

6 Hunan Provincial Key Laboratory of Forestry Biotechnology, Central South University of Forestry and Technology, Changsha 410004, China

7 School of Life Sciences, Xinyang Normal University, Xinyang 464000, China; l473973649@foxmail.com

* Correspondence: louwj@qibebt.ac.cn (W.L.); lvxf@qibebt.ac.cn (X.L.)

\# These authors contributed equally to this work.

Supplemental data

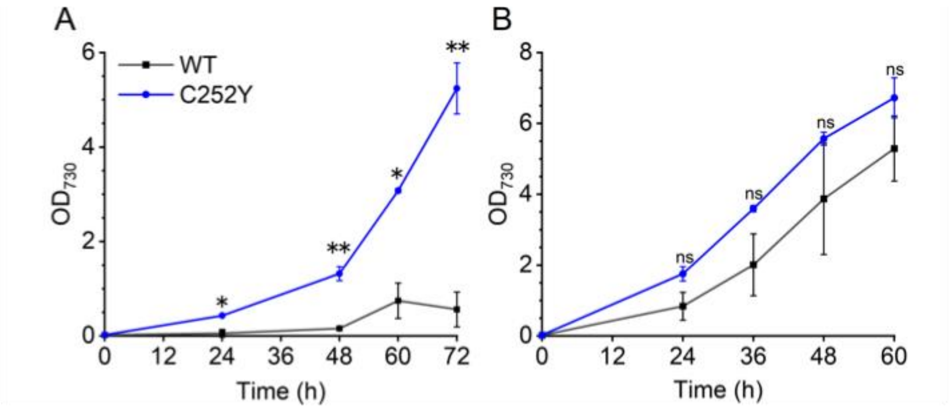

Figure S1. Growth of *Synechococcus elongatus* PCC 7942 (*Sye*7942) strains under various HLHT conditions. Growth of the wild type *Sye*7942, C252Y mutant, and ATPAOE strains were cultured in liquid BG11 medium at (**A**) 41 °C with 2000 μmol photons m^{-2} s^{-1} illumination; (**B**) 41 °C with 1500 μmol photons m^{-2} s^{-1} illumination. WT, wild type *Sye*7942; C252Y, the C252Y mutant. Stars, significant differences ($P < 0.05$); two stars, highly significant ($P < 0.01$); three stars, extremely significant ($P < 0.001$); ns, no significant difference. Error bars indicate standard deviations ($n = 3$).

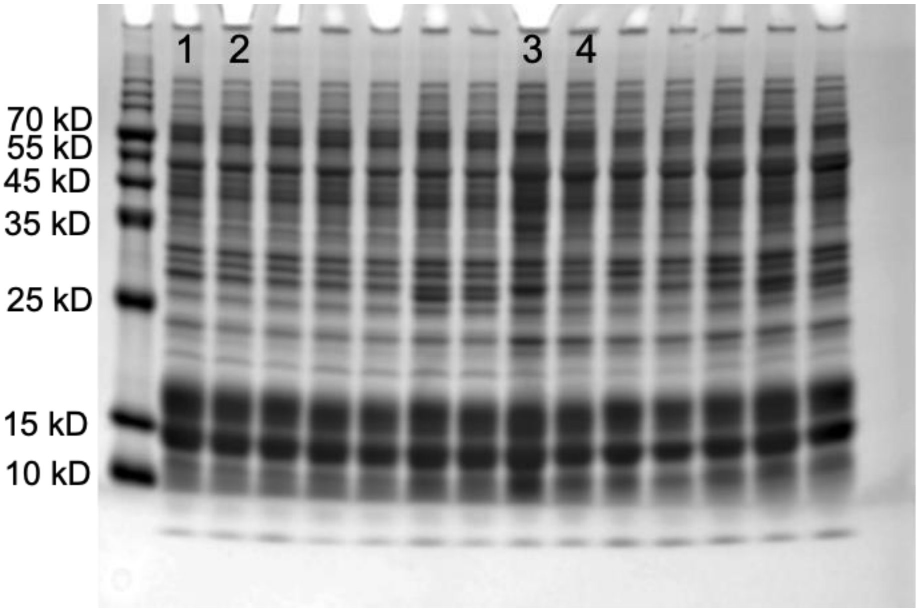

Figure S2. SDS-PAGE analysis of the total protein samples from *Sye*7942 strains grown under NTNL and HLHT conditions. The wild type *Sye*7942, C252Y mutant, and ATPAOE strains were grown under normal conditions (NTNL; 30 °C, 280 μmol photons m^{-2} s^{-1}) and high light and temperature conditions (HLHT; 40 °C, 2000 μmol photons m^{-2} s^{-1}). Total proteins were isolated from these samples, and each sample containing 1.6 μg *Chl* a was loaded and analyzed by SDS-PAGE. Line 1 and 3, wild type *Sye*7942; line 2 and 4, the C252Y mutant strain; line 1 and 2, grown under NLNT conditions; line 3 and 4, grown under HLHT conditions.

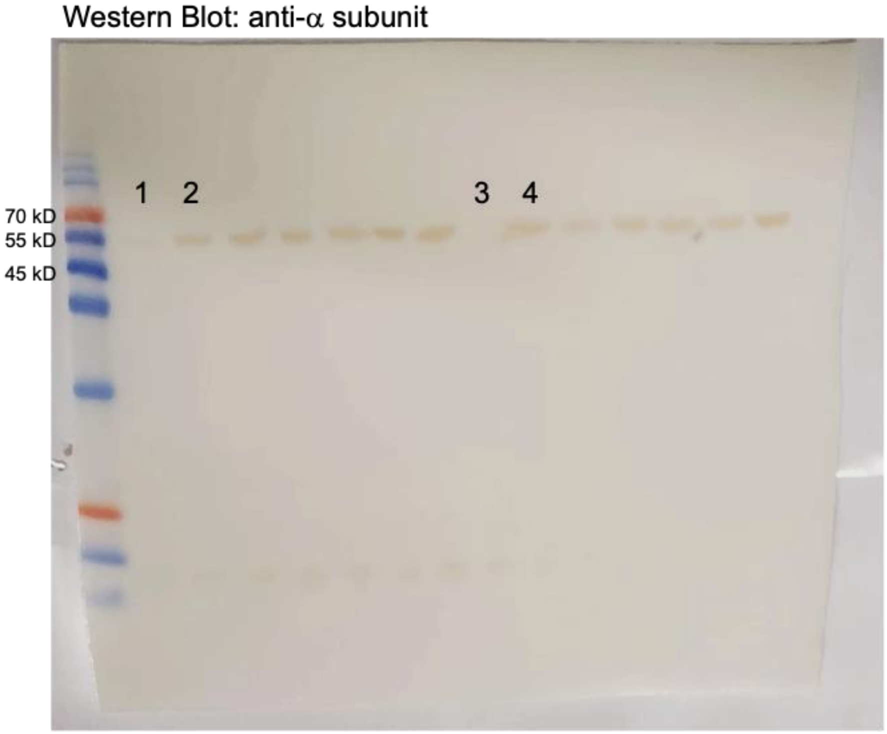

Figure S3. The original figure of figure 2, anti-α subunit. Line 1 and 3, wild type *Sye*7942; line 2 and 4, the C252Y mutant; line 1 and 2, grown under NLNT conditions (30 ^{o}C, 280 μmol photons m^{-2} s^{-1}); line 3 and 4, grown under HLHT conditions (40 ^{o}C, 2000 μmol photons m^{-2} s^{-1}).

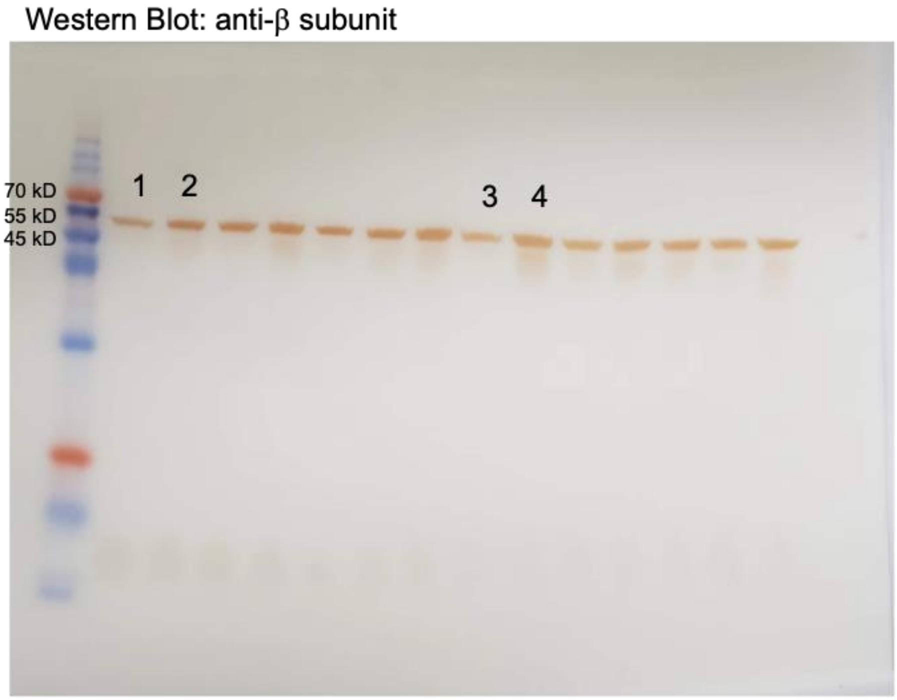

Figure S4. The original figure of figure 2, anti-β subunit. Line 1 and 3, wild type *Sye*7942; line 2 and 4, the C252Y mutant; line 1 and 2, grown under NLNT conditions (30 ^{o}C, 280 μmol photons m^{-2} s^{-1}); line 3 and 4, grown under HLHT conditions (40 ^{o}C, 2000 μmol photons m^{-2} s^{-1}).

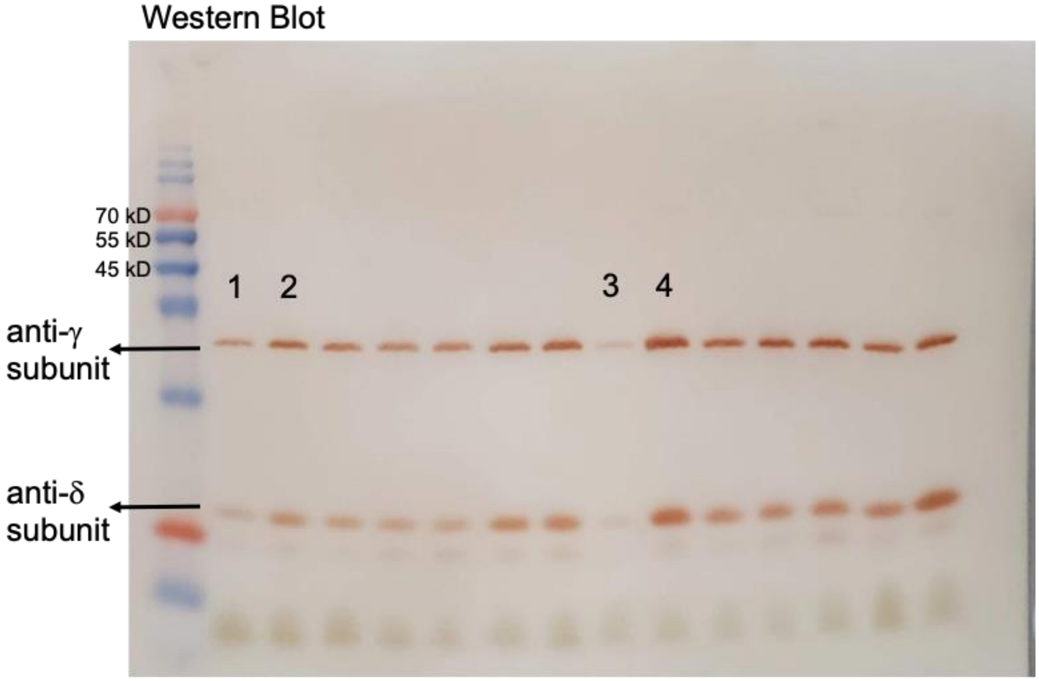

Figure S5. The original figure of figure 2, anti-β and δ subunit. Line 1 and 3, wild type *Sye*7942; line 2 and 4, the C252Y mutant; line 1 and 2, grown under NLNT conditions (30 ^{o}C, 280 μmol photons m^{-2} s^{-1}); line 3 and 4, grown under HLHT conditions (40 ^{o}C, 2000 μmol photons m^{-2} s^{-1}).

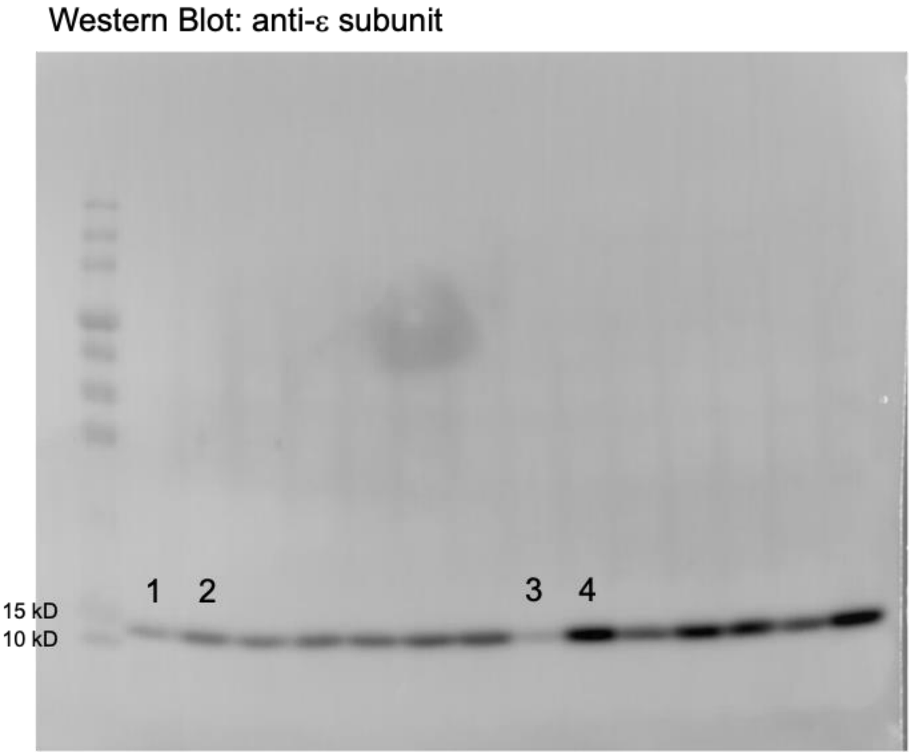

Figure S6. The original figure of figure 2, anti-ε subunit. Line 1 and 3, wild type *Sye*7942; line 2 and 4, the C252Y mutant; line 1 and 2, grown under NLNT conditions (30 °C, 280 μmol photons m^{-2} s^{-1}); line 3 and 4, grown under HLHT conditions (40 °C, 2000 μmol photons m^{-2} s^{-1}).

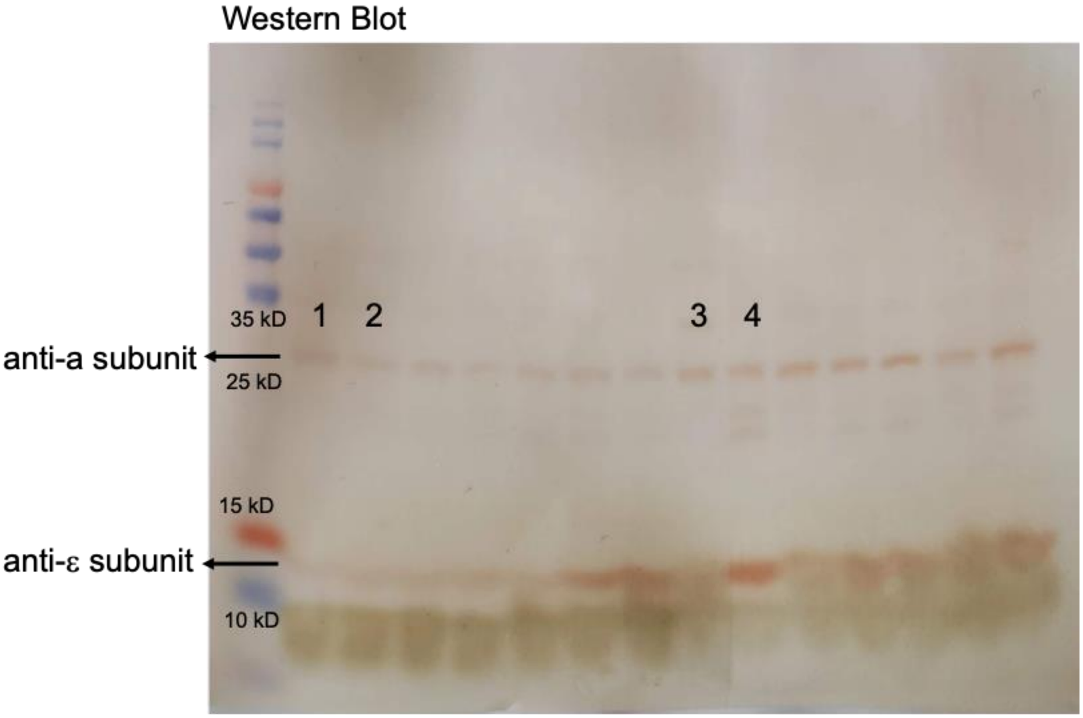

Figure S7. The original figure of figure 2, anti-a subunit. Line 1 and 3, wild type *Sye*7942; line 2 and 4, the C252Y mutant; line 1 and 2, grown under NLNT conditions (30 °C, 280 μmol photons m^{-2} s^{-1}); line 3 and 4, grown under HLHT conditions (40 °C, 2000 μmol photons m^{-2} s^{-1}).

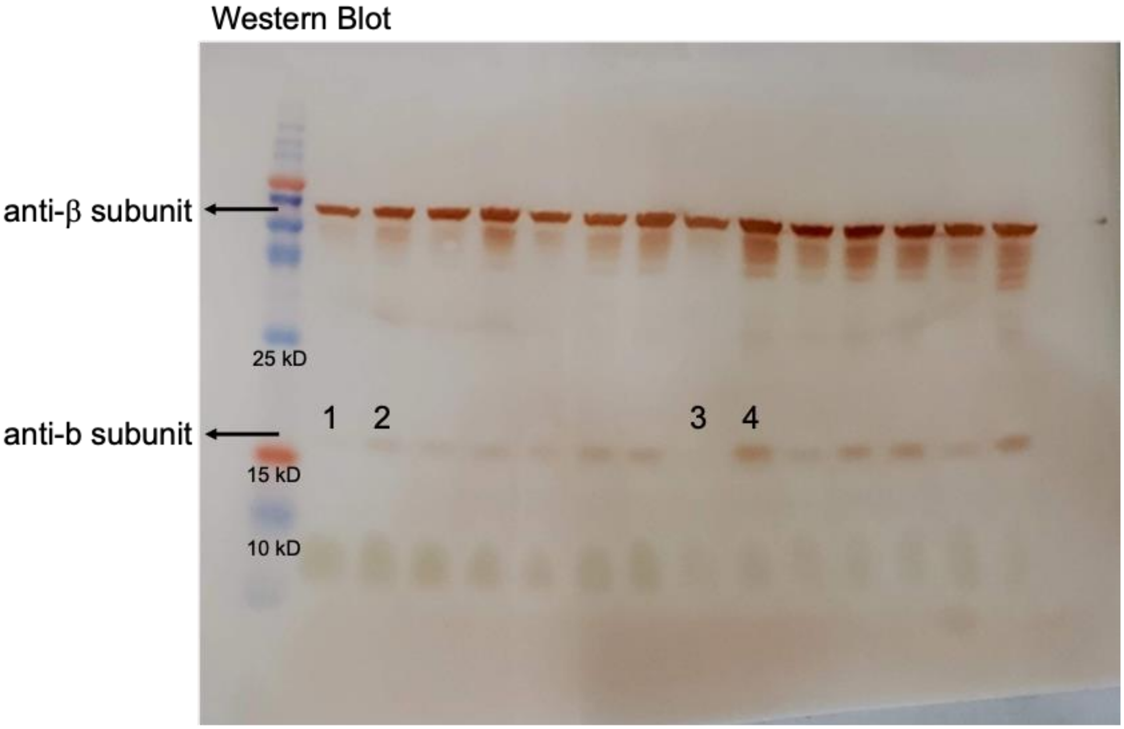

Figure S8. The original figure of figure 2, anti-b subunit. Line 1 and 3, wild type *Sye*7942; line 2 and 4, the C252Y mutant; line 1 and 2, grown under NLNT conditions (30 °C, 280 μmol photons m^{-2} s^{-1}); line 3 and 4, grown under HLHT conditions (40 °C, 2000 μmol photons m^{-2} s^{-1}).

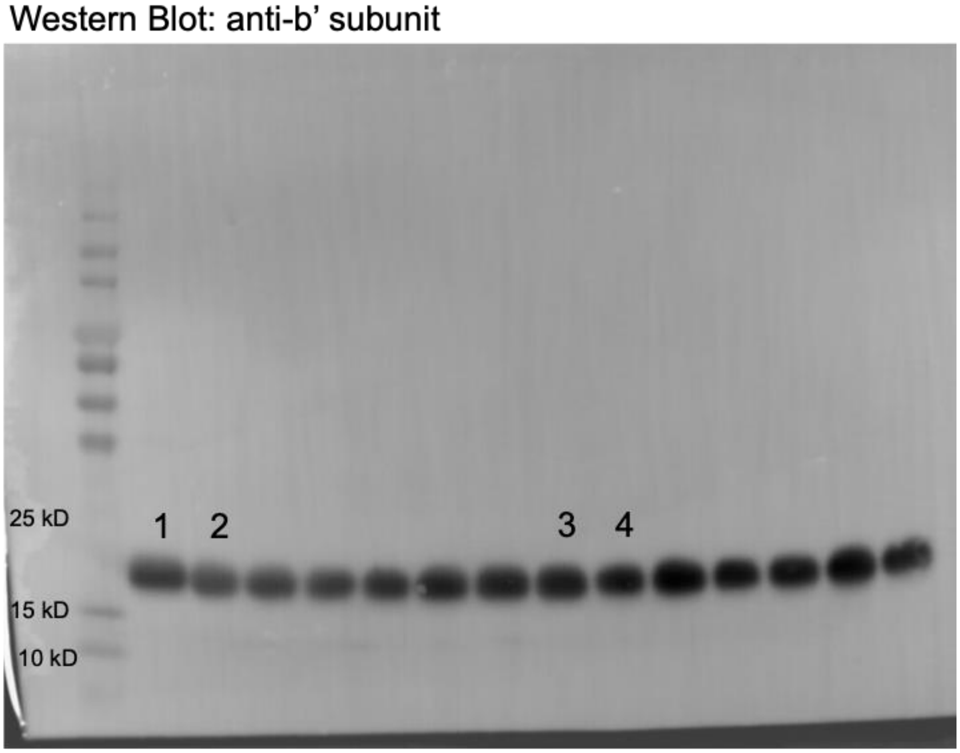

Figure S9. The original figure of figure 2, anti-b′ subunit. Line 1 and 3, wild type *Sye*7942; line 2 and 4, the C252Y mutant; line 1 and 2, grown under NLNT conditions (30 ^{o}C, 280 μmol photons m^{-2} s^{-1}); line 3 and 4, grown under HLHT conditions (40 ^{o}C, 2000 μmol photons m^{-2} s^{-1}).

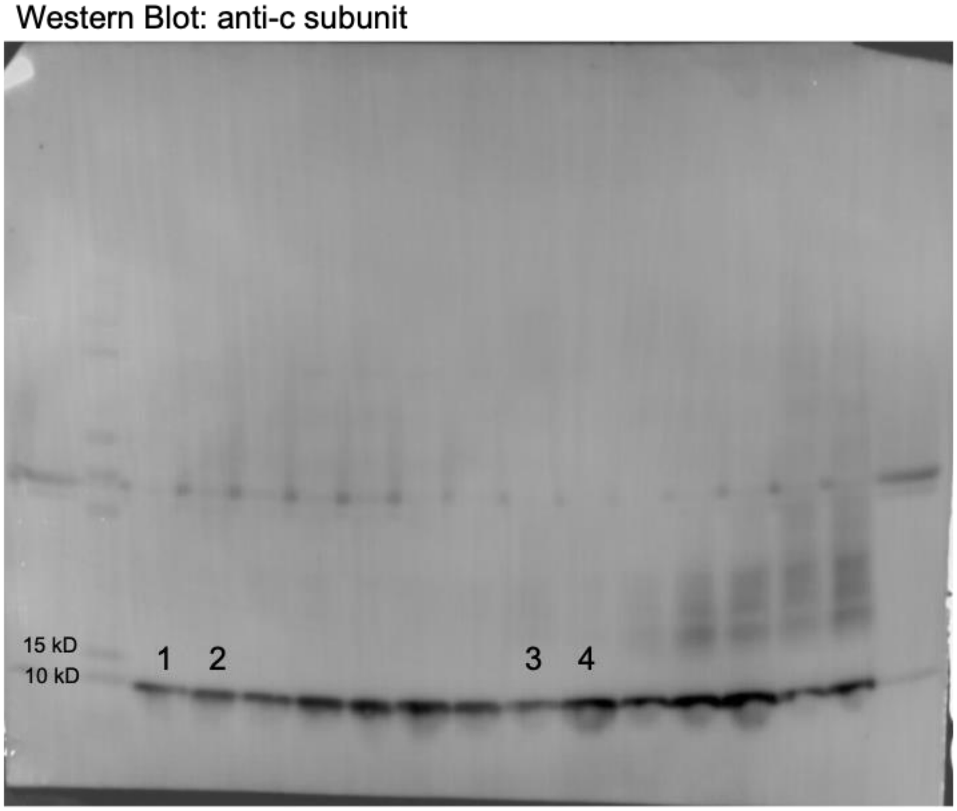

Figure S10. The original figure of figure 2, anti-c subunit. Line 1 and 3, wild type *Sye*7942; line 2 and 4, the C252Y mutant; line 1 and 2, grown under NLNT conditions (30 °C, 280 μmol photons m^{-2} s^{-1}); line 3 and 4, grown under HLHT conditions (40 °C, 2000 μmol photons m^{-2} s^{-1}).

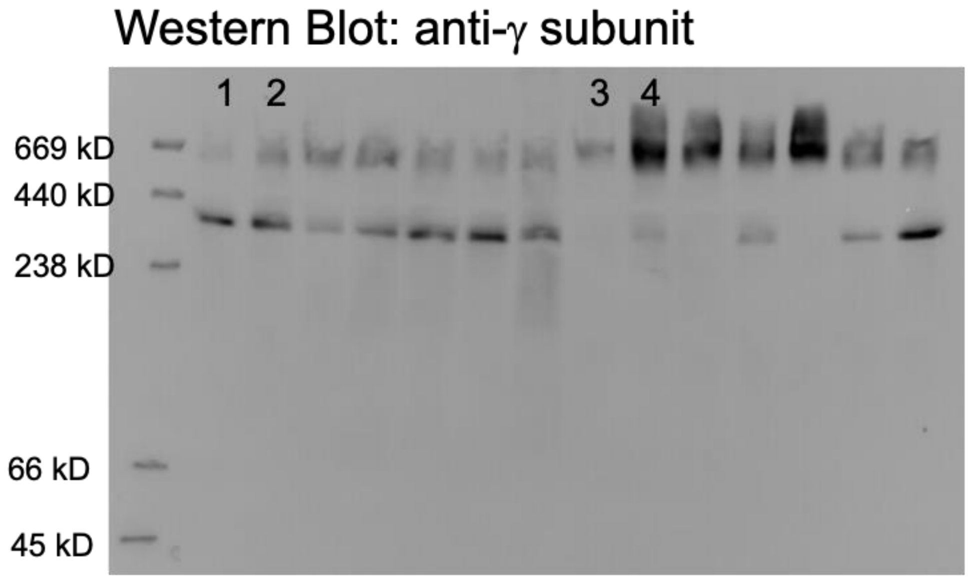

Figure S11. The original figure of figure 3C, anti-γ subunit. Line 1 and 3, wild type *Sye*7942; line 2 and 4, the C252Y mutant; line 1 and 2, grown under NLNT conditions (30 °C, 280 µmol photons m^{-2} s^{-1}); line 3 and 4, grown under HLHT conditions (40 °C, 2000 µmol photons m^{-2} s^{-1}).

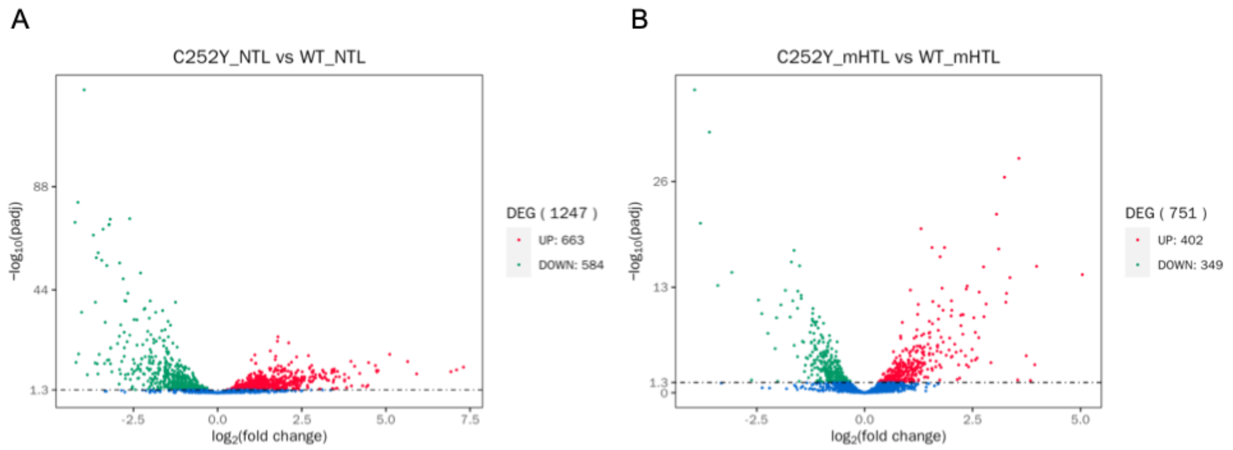

Figure S12. Volcano plot comparing gene expression differences between the wild type and C252Y mutant. The transcriptomes of the wild type and C252Y mutant were analyzed by RNA-seq under (**A**) NTNL (30 ^{o}C, 280 μmol photons m^{-2} s^{-1}) and (**B**) HLHT (40 ^{o}C, 2000 μmol photons m^{-2} s^{-1}) conditions. Genes with an adjusted P-value < 0.05 were assigned as differentially expressed. NTL, normal temperature and light (NTNL); mHTL, high light and temperature (HLHT); WT, wild type *Sye*7942; C252Y, the C252Y mutant.

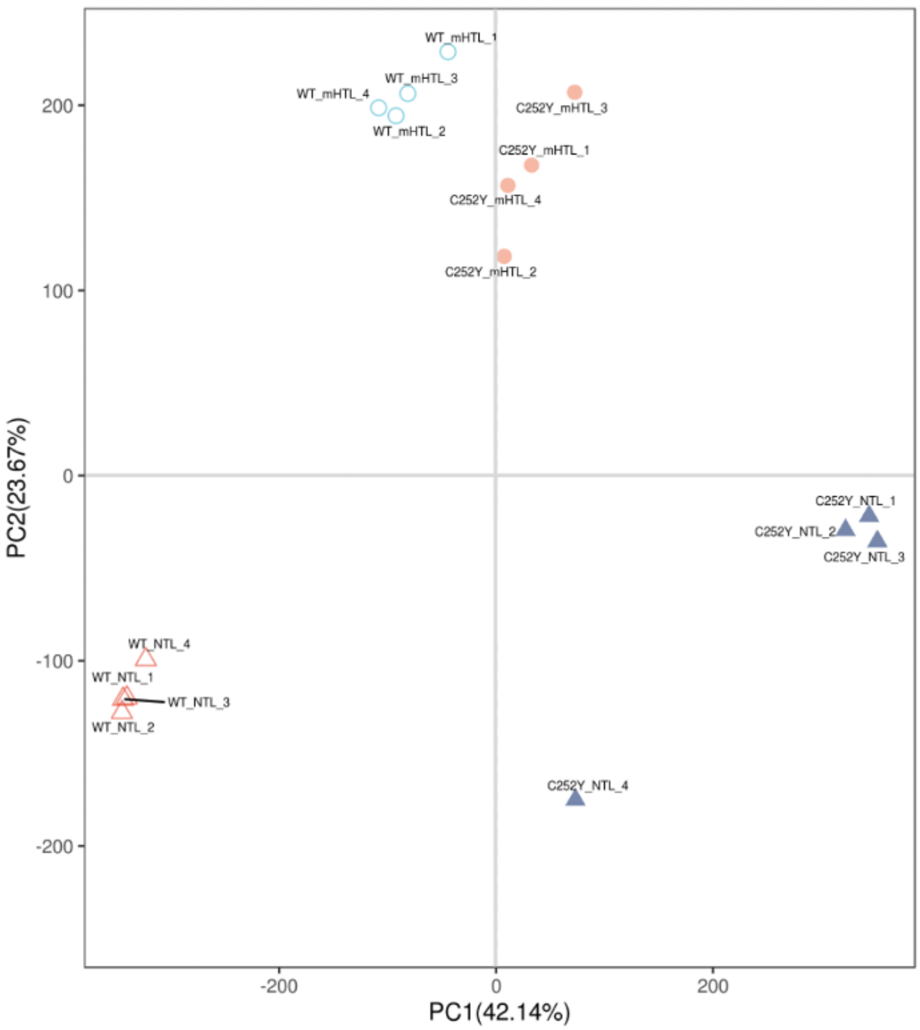

Figure S13. Principal component analysis (PCA) of RNA sequencing data. NTL, normal temperature normal light (NTNL; 30 °C, 280 μmol photons m^{-2} s^{-1}); mHTL; high light high temperature (HLHT; 40 °C, 2000 μmol photons m^{-2} s^{-1}); orange tringle, the wild type grown under NTNL conditions; light blue circle, the wild type grown under HLHT conditions; dark blue solid tringle, the C252Y mutant grown under NTNL conditions; orange solid circle, the C252Y mutant grown under HLHT conditions.

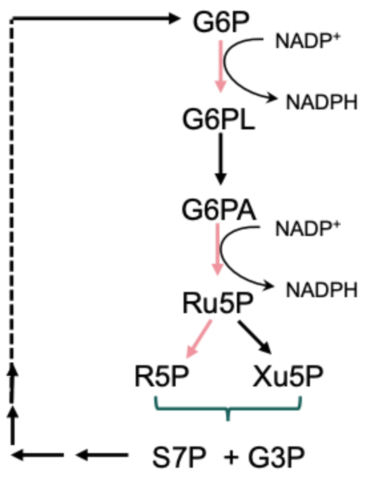

Figure S14. Changes in gene expression of the oxidative phase of the pentose phosphate pathway (OPP) genes due to the C252Y mutation in HLHT conditions (the mutant vs wild type; 40 °C, 2000 μmol photons m^{-2} s^{-1}). Genes with a transcription fold change log2 > 0.5 and P < 0.05 were included in the analysis. Enzymes that upregulated were indicated by red arrows, consistently expressed enzymes by black arrows, and downregulated enzymes by green arrows. Abbreviations: G6P, G6P, glucose 6-phosphate; G6PL, 6-phosphoglucono-δ-lactone; G6PA, 6-phosphogluconate; Ru5P, ribulose 5-phosphate; R5P, ribose 5-phosphate; Xu5P, xylulose 5-phosphate; S7P, sedoheptulose 7-phosphate; G3P, glycogen 3-phosphate.

Table S1. Qualification the Western blot data. Western blot data was analyzed using ImageJ to qualify the protein levels, which were then normalized to the protein levels of the wild type grown under normal light normal temperature conditions.

ATP synthase subunits	NTNL		HLHT	
	WT	C252Y	WT	C252Y
α	1.0	2.7	0.6	3.3
β	1.0	1.5	1.0	1.3
γ	1.0	1.9	0.5	3.6
ε	1.0	2.1	0.8	6.1
δ	1.0	2.2	0.5	4.7
a	1.0	1.1	2.4	2.5
b	1.0	2.4	1.2	8.7
b′	1.0	0.9	1.1	1.2
c	1.0	1.0	1.0	1.4
F_1-subcomplex	1.0	1.2	0	0.2
ATP synthase	1.0	3.9	4.9	29.6

NTNL, normal temperature normal light (30 °C, 280 μmol photons m^{-2} s^{-1}); HLHT, high light high temperature (40 °C, 2000 μmol photons m^{-2} s^{-1}); WT, wild type *Sye*7942; C252Y, the C252Y mutant.

Table S2. Differential gene expression analysis of the pentose phosphate pathways in the C252Y mutant versus the wild type under HLHT conditions (40 °C, 2000 μmol photons m^{-2} s^{-1}): fold change log2 > 0.5, $P < 0.05$.

Gene ID	Gene functional identification	fold change (times)
Pentose phosphate pathway		
SYNPCC7942_RS11865	glucose-6-phosphate dehydrogenase; zwf	2.8
SYNPCC7942_RS11870	class 1 fructose-bisphosphatase; fbp	2.3
SYNPCC7942_RS02970	ribose-5-phosphate isomerase; rpiA	2.6
SYNPCC7942_RS10295	glucose-6-phosphate isomerase	1.6
SYNPCC7942_RS11675	transaldolase	-1.6
SYNPCC7942_RS00195	NADP-dependent phosphogluconate dehydrogenase; gndA	1.7

Table S3. Primers used in this study.

Plasmid/primers	Primer sequence (5′→ 3′)
**pWL8**	
qd-oWL41	TCAAACGCTGACTAGACCACCCG
qd-oWL42	GTCTAGTCAGCGTTTGACCTGAATTGACTCTCTTCCGGGC
qd-oWL45	AAGGAGATATACCATGGCTCCGCAACCCTTCCTTCC
qd-oWL46	CATGGTATATCTCCTTCTTAAAGTTAAACAAAATTATTTCTAGAGG
qd-oWL47	TGGTGATGGCTGCTGCCCTAGCCGTTCAGCGCTTCGGC
qd-oWL48	GGCAGCAGCCATCACCATCATC
qd-oWL49	
oYY25	ACATATGCATCACCATCATCACCACGTCACCACTACTGAGAAGACAAATATTG G
oYY26	CCATCTTAGTATATTAGTTAAGTATAAGAAGGAGATATACATATGCATCACCAT CATCACC
**pWL8**	
qd-oWL80	TTTGCCTTTGTACATAAAGTACTCGGCGATCG
qd-oWL81	TTTATGTACAAAGGCAAAGCCACCTTGGTC

Table S4. Sequencing data statistics.

Sample name	Raw reads	Raw bases	Clean reads	Clean bases	Error rate	Q20	Q30	GC content
WT_NTL_1	22543936	3.38G	18956684	2.84G	0.02%	98.87%	96.12%	47.58%
WT_NTL_2	23731854	3.55G	19384482	2.91G	0.02%	98.65%	95.57%	48.42%
WT_NTL_3	23235014	3.48G	18161124	2.72G	0.02%	98.75%	95.75%	48.56%
WT_NTL_4	22273782	3.34G	18221312	2.73G	0.02%	98.67%	95.64%	48.51%
WT_mHTL_1	22731292	3.4G	18405148	2.76G	0.02%	98.36%	94.97%	52.20%
WT_mHTL_2	22632410	3.39G	18667946	2.8G	0.02%	98.46%	95.20%	51.78%
WT_mHTL_3	23343066	3.5G	18895984	2.83G	0.02%	98.66%	95.69%	51.99%
WT_mHTL_4	19635028	2.94G	14912114	2.24G	0.02%	98.51%	95.32%	51.65%
C252Y_NTL_1	24298904	3.64G	19734694	2.96G	0.02%	98.41%	95.14%	53.23%
C252Y_NTL_2	23228868	3.48G	18658278	2.8G	0.02%	98.39%	95.03%	52.65%
C252Y_NTL_3	23726228	3.55G	19701598	2.96G	0.02%	98.35%	94.97%	53.18%
C252Y_NTL_4	20715952	3.1G	17505306	2.63G	0.02%	98.83%	96.07%	49.31%
C252Y_mHTL_1	19789632	2.96G	15386686	2.31G	0.02%	98.48%	95.27%	51.49%
C252Y_mHTL_2	21103324	3.16G	16586572	2.49G	0.02%	98.72%	95.84%	51.52%
C252Y_mHTL_3	29346024	4.4G	22831956	3.42G	0.02%	98.49%	95.30%	52.66%
C252Y_mHTL_4	22040212	3.3G	16769640	2.52G	0.02%	98.52%	95.34%	51.24%

NTL, normal temperature normal light (NTNL; 30 ^{o}C, 280 μmol photons m^{-2} s^{-1}); mHTL, high light high temperature (HLHT; 40 ^{o}C, 2000 μmol photons m^{-2} s^{-1}); WT, wild type *Sye*7942; C252Y, the C252Y mutant; Error rate, average error rate of clean reads; Q20、Q30, percentage of bases with Phred quality scores above 20 and 30 in relation to the total number of bases in clean data.
